# Characterizing Adolescents' Dietary Intake by Taste: Results From the UK National Diet and Nutrition Survey

**DOI:** 10.3389/fnut.2022.893643

**Published:** 2022-06-20

**Authors:** Areej Bawajeeh, Michael A. Zulyniak, Charlotte E. L. Evans, Janet E. Cade

**Affiliations:** ^1^Nutritional Epidemiology Group, School of Food Science and Nutrition, University of Leeds, Leeds, United Kingdom; ^2^Department of Food and Nutrition, Faculty of Human Sciences and Design, King Abdulaziz University, Jeddah, Saudi Arabia

**Keywords:** taste, dietary taste, NDNS, adolescents, taste perception

## Abstract

The taste of foods is a key factor for adolescents' food choices and intakes, yet, exploring taste characteristics of adolescents' diet is limited. Using food records for 284 adolescents (10–19 years old) from the National Diet and Nutrition Survey (NDNS), year 9 (2016–2017), we classified diets according to taste. Tastes for each food consumed were generated from a previous survey that asked participants to allocate one main taste to each food. Responses from that survey were processed and included in a Hierarchical Cluster Analysis (HCA) to identify taste clusters. The resulting tastes were then applied to the adolescents' food records in the NDNS. For each individual, the total weight of food per day for each taste was calculated. A linear regression model was used to explore dietary intakes from each taste. Findings reveal that adolescents' daily energy intake was highest (34%) from foods that taste sweet. Sweet foods were the main calorie contributors at breakfast and daytime snacking, while energy intake from neutral-tasting foods was higher at lunch and dinner. Sweet food intake was significantly positively associated with higher energy, sugar, and fat intakes. For each percentage increase in sweet foods, energy increased by 10 kcal/d (95% CI 6, 15; P < 0.01). Savory food intake was lower in carbohydrates and sugars; with neutral food consumption inversely associated with energy, carbohydrate, sugars, saturated and total fat. Higher salty food intake was linked to higher saturated fat as well as sodium consumption. Sweet and neutral foods dominate the UK adolescent diet, followed by savory tastes. Balancing the contributions of different tasting foods could assist in improving adolescent diet quality.

## Introduction

The taste of foods has been reported to be an important predictor in food choice decisions, independently of a range of factors, such as cost, availability, food appearance, hunger, socio-environmental and socio-economic characteristics that influence food choices and intake (1–3). Individual variations in taste perception may lead to differences in dietary intake which in turn influences nutritional status (4, 5). The sense of taste (i.e., gustation) is a sensory modality that allows humans to perceive the basic tastes in foods (sweet, salty, sour, bitter, and savory/umami) when the substances in foods interact and stimulate taste receptor cells on the tongue (6). Early sweet taste preferences in humans are innate; with salty taste preference starting during the first few months after birth, while bitter and sour tastes are less attractive (7). However, these innate preferences are not stable throughout life. Children are likely to have taste preferences that are comparable to those they experienced in their early life (8); however, observing adults' pleasure in eating vegetables through enjoyable comments and facial expressions can motivate a young child's curiosity and overcome their refusal of bitter vegetables, like broccoli (9). As the child enters adolescence, parental influences on their child's taste preferences in relation to food choices and intake is less effective (9, 10).

In sensory studies, individuals' taste perception can be assessed by subjectively nominating the perceived taste quality and/or intensity (11). This is known as phenotype assessment and has been widely used in sensory studies aiming to identify individuals' perceptions and classify their actual experience of tastes. A number of sensory studies (i.e., taste perception and/or preference studies) have been conducted in relation to food choice and dietary intake in different age groups (12–17). Sweet, salty and savory tastes have been shown to influence energy intake (18). In our previous systematic review of adolescents' taste perception and food choices, we found that perceived bitterness in cruciferous vegetables (i.e., broccoli, cabbage, Brussels sprouts and cauliflower) was negatively associated with intake and preferences and positively associated with energy intake. However, this was not consistent due to variations in the taste assessment among the studies (12); likewise in adults (19–21). This inconsistency may be due to variations in the taste assessment where studies have tested this relationship using liquid solutions of taste samples and/or limited individual food items as references to evaluate the influence of individuals' taste perceptions and/or preferences on selected dietary outcomes.

Studies assessing the taste perceptions of foods consumed in a real-world context integrated with food composition data are limited to a small number of studies (22–26). An innovative “*in-home*” method was used to create a food-taste database for foods that were frequently consumed by the study participants (24). Another study quantified the taste intensity of fifty frequently consumed Dutch foods (25), while an Australian study quantified a sensory profile of a wider range of food intake data from a national survey (23). None of these studies assessed how taste influenced their populations' dietary intake. van Langeveld et al. studied Dutch adults' dietary taste patterns using a taste profile generated for food intake data reported in the Dutch National Food Consumption Survey (26). However, the authors only assessed taste contributions to energy intake. In an earlier small study, researchers studied the association between taste characteristics of foods and dietary intake of 41 UK adults. The study used dietary intake records of participants who were asked to assign one predominant taste for the reported consumed foods (22). This study also only focused on energy intake by taste, identifying differences between obese and non-obese adults, but such a study is absent in adolescents. Findings from the existing literature indicate that taste is not just a sense that motivates people's food choices and consumption, but it can imply and signal calories and nutrients in foods. Since adolescents have indicated taste as an important factor when selecting and consuming foods, how taste links to intakes needs to be explored.

Adolescence is a critical phase of growth and development transitioning from childhood to adulthood (27). Thus, healthy eating and good nutrition are required during this period to meet growth needs; however, one way in which adolescents assert their independence and autonomy is in relation to food choices (28), which may not always be healthy. Food choices among adolescents have been found to be predominantly based on food taste, with a greater consumption associated with foods that satisfy their preferences (29–32). They often consume more sweetened drinks and fast foods but lower intakes of fruits and vegetables (33). This may be because the sugar, salt, and fat content of these drinks and foods provide pleasant tastes (34) while vegetables are often related to unpleasant bitterness and sourness (35). Dietary intake that is driven by individuals' taste preference may be related to future health risks (36), especially, during adolescence as a critical period of development. Therefore, it is important to understand the relationships between taste, dietary habits, and nutritional intakes in this age group (12). Thus, the purpose of this study was to characterize the taste of foods using adolescents' food records from the National Diet and Nutrition Survey (NDNS) and to assess the taste characteristics in relation to food and nutrient intakes of UK adolescents.

## Methods

**Figure 1:**
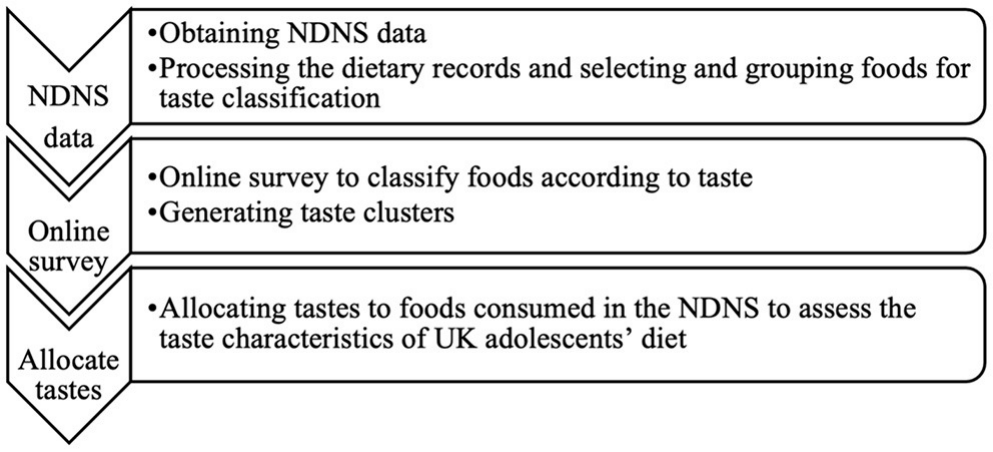
Study outline to classify food tastes in UK adolescents.

This study used food intake data from 284 adolescents (girls = 144 and boys = 140) aged 10–19 years old in the UK National Diet and Nutrition Survey (NDNS) rolling program, year 9 (2016/2017). The NDNS is an annual cross-sectional survey assessing dietary intake and nutritional status of a UK representative sample aged 1.5 + years who are randomly recruited based on postcode. The dietary data are collected using the estimated food record method. Parents/carers of adolescents aged ≤ 12 years are asked to help their children to complete the diary, while those who are ≥13 years completed their diary themselves. Participants are asked to keep a record of everything they consumed with estimated quantities of consumption for 4 consecutive days. A check-up visit by trained interviewers is arranged to review the diary for any clarification needed. Food items are then categorized into main and sub food groups and assigned a food code and name. In the current study, the detailed food record dataset “Food Level Dietary Data” was used for grouping foods to support food taste classification. Survey details and methodology of the NDNS have been reported elsewhere (37). Figure 1 illustrates the steps undertaken in the current study.

### Processing Dietary Data and Selecting Foods for Taste Classification Survey

Foods in the NDNS diaries were grouped according to how foods may be consumed. This step was necessary to harmonize the data since some composite dishes had been coded as separate ingredients and some coded as single composite items. To do this we used the following approach:

Food items that were ingredients of composite foods and were linked together with one code for that composite dish. For example, ingredients of “Chicken curry” (e.g., chicken, curry sauce, onion… etc.) were linked under one new code.

Dry/powdered items (e.g., instant coffee, drinking chocolate) and concentrated juices where water or another diluent was listed for the same reported mealtime were also combined.

Where the food items could be eaten separately, they were allocated tastes individually and not combined with one coded composite item. For example, a cheese and bacon sandwich was retained as bread, cheese, and bacon separately.

More than 1,743 different food items were identified as having been consumed by the adolescents in the NDNS records. These food items were grouped with similar items to create a manageable list of foods for inclusion in the online questionnaire, limiting the burden on participants.

For inclusion in the questionnaire, foods were identified based on consumption frequency, contribution to energy, and contribution to taste (e.g., salad dressing, ketchup). Foods were grouped into food groups using the NDNS main and subsidiary food groups with further considerations such as sugar/salt content (e.g., sweet biscuits or salty biscuits were kept separate) and fruit and vegetable types known to be sweet, sour, or bitter. Supplementary Table S1 in the supplementary shows examples of the grouped food list under the main and subsidiary food groups. These were checked and agreed by the members of the research team.

### Developing an Online Food-Taste Classification Survey

The list of foods was used in an online survey asking a sample of regular consumers to classify the taste of the foods. Ethics approval for the online survey was obtained from the University of Leeds MEEC 19-039.

Participants were asked to assign one main taste to each food. Taste choices given were sweet, salty, sour, bitter, savory/umami, neutral, or never tried, with an explanation provided for each taste (Supplementary Table S2). An initial list of 239 food items was piloted with 19 individuals to identify foods where everyone allocated the same taste to further limit the list. Following this pilot test, 55 foods were removed (Supplementary Table S3) which had a very high level of agreement on taste classification. For example, cakes, fruit yogurt, and unsweetened apple juice were allocated by all in the pilot test as sweet tasting. A final list of 184 food items to be rated for taste was generated. A convenience sample was used, distributing the survey online *via* Facebook and Twitter platforms as well as to individuals known to the researchers. Due to ethical considerations, only respondents aged 18 years and above were allowed to complete the survey. Whilst it is possible that there are some taste changes between adolescence and adulthood, these are likely to be in terms of taste intensity and concentration preference, rather than detection or sensation of taste (38, 39). To minimize participant burden, the food lists were divided into three parts (40) and participants were asked to complete one part with an option to voluntarily complete the rest.

Taste classification of our participants was tested through concurrent validity with trained panel data by checking responses from the taste classification survey against taste profiles developed by trained panelists from previous publications in the literature (26, 41, 42). A total of 123 food items were available for comparison checking. As illustrated in Supplementary Table S4, there was 84% agreement (*n* = 103), 7% disagreement and 9% neutral.

## Data Analysis

Following survey completion, for each food item, the percentage of respondents choosing each taste was included in a Hierarchical Cluster Analysis (HCA) using Python Software Foundation version 3.9 to identify taste clusters. The “never tried” responses were excluded from the analysis. The number of clusters was determined based on the dendrogram and assessment of the scree plot (43). The identified tastes were allocated to individual food items in the NDNS. For example, all cake types that were reported in the NDNS were grouped as “cakes” in our grouped food list used in the online questionnaire. Then from the HCA, “cakes” were classified under the sweet taste cluster. Thus, all individual codes for cakes in the NDNS were allocated a sweet taste.

A specific taste was allocated to each food consumed in the NDNS. Then for each individual, foods contributing to each taste group were summed and the proportion of the weight of the food consumed per day was then calculated for each taste by dividing the weight of foods in each taste group by the total weight of food consumed.

Linear regression modeling was used to compare the mean differences of daily energy intake from each taste cluster by gender, age group (younger adolescents aged 10–14 years and older adolescents aged 15–19 years), and BMI categories (normal weight, overweight and obese). Repeated measures ANOVA, with Bonferroni *post-hoc* test, was used to compare the mean difference of energy intake from each taste cluster between eating occasions during the day. Eating occasions were chosen according to the time of day as in a previous study using the NDNS data. Time frames are 06.00 to 08.59 am (breakfast), 12.00 noon to 1.59 pm (lunch) and 17.00 pm to 19.59 pm (dinner). Snacking is defined as eating occasions outside meal times (44).

The final analysis explored characteristics of the adolescents' dietary intakes by taste. A test for trend was conducted using the percentage of foods from each taste group (exposure) as continuous variables by food and nutrient intakes (outcomes) in linear regression modeling. The percentage of food weight for each taste was split into quintiles to illustrate the direction of effect. The sour taste cluster was presented as only two categories (consumers and non-consumers) due to the high proportion of non-consumers of sour foods.

Weighting to adjust for non-response in the NDNS was applied in all analyses using weights provided for the NDNS (37). Statistical significance was assigned to a *P*-value < 0.05 for all tests. The statistical analysis was performed using STATA statistical software version 16.1.

## Results

### Online Food-Taste Classification Survey

In total 209 responses (162 females, 44 males & 3 not known) were obtained. Around 90% of the survey respondents were British/white European, and their age ranged from 20 to 70 years, with the majority being between 40 and 59 years old. The HCA grouped the 184 foods/food groups in the questionnaire into six main taste clusters (sweet, salty, sour, bitter, savory, and neutral). Applying these tastes to the 1743 individual food codes in the diaries resulted in the following: 40% of foods (*n* = 703) were sweet, 27% (*n* = 463) were neutral, 20% (*n* = 346) were savory, 7% (*n* = 115) were salty, 4% (*n* = 77) were bitter and 2% (*n* = 39) were sour.

Foods that mostly contributed to the sweet taste cluster were sweet snacks (sweet biscuits, chocolates and candies), desserts (cakes, sweet pastries, and pies) sweetened beverages, dairy products, and fruit. Foods characterized as neutral tasting included potatoes, bread, white fish, and some vegetables. Savory tastes included meats and poultry products and flavored/spiced foods. Foods with a high salt content were, as expected, included in the salty taste, with the top contribution coming from snacks (crisps, salty biscuits and crackers). Most of the foods contributing to bitter taste came from vegetables known for their bitter taste, such as Brussel sprouts, cabbage, coffee, and tea. Some fruits (e.g., kiwi and other fruit that have some sourness) and salad dressing were characterized as sour-tasting items. Supplementary Table S5 in the supplementary illustrates common examples of foods items contributed to each taste.

### Contribution of the Identified Tastes to the UK Adolescents' Daily Energy Intake From the NDNS

Table 1 illustrates adolescents' energy intake from each taste stratified by sample characteristics and eating occasions. The major contributions to adolescents' daily energy intakes were from sweet-tasting foods (34%) 558 kcal/d (95%CI 516, 599), neutral-tasting foods (34%) 556 kcal/d (95%CI 521, 592), and savory-tasting foods (21%) 334 kcal (95%CI 307, 362), salty, bitter, and sour tasting foods provided much smaller energy contributions.

**Table 1:** Adolescents' energy intake (kcal/d) as a total and from each taste stratified by sample characteristics and eating occasions.

		**Energy intake** **(kcal/ day)** **Mean (95%CI)**	**Taste contribution to energy intake (kcal/d)**					
			**Mean (95%CI) & (% of total energy)**					
			**Sweet**	**Neutral**	**Savory**	**Salty**	**Bitter**	**Sour**
	Total sample (*n* = 284)	1,626 (1,565, 1,688)	558 (516, 599) (34%)	556 (521, 592) (34%)	334 (307, 362) (21%)	163 (146, 181) (10%)	10 (6,13) (1%)	5 (2,7) (<1%)
Gender	Boys (*n* = 140)	1,729 (1,651, 1,808)	614 (551, 677) (36%)	581 (540, 623) (34%)	349 (309, 389) (20%)	171 (148, 195) (10%)	10 (5,16) (1%)	3 (1,5) (<1%)
	Girls (*n* = 144)	1,523 (1,427, 1,616)	501 (450, 552) (33%)	531 (473, 590) (35%)	320 (282, 357) (21%)	155 (129, 181) (10%)	8 (4,13) (1%)	6 (2,11) (<1%)
Age group	10–14 years (*n* =174)	1,596 (1,516, 1,675)	586 (530, 643) (37%)	528 (477, 579) (33%)	293 (263, 323) (18%)	181 (156, 205) (11%)	4 (1,7) (<1%)	3 (2,5) (<1%)
	15–19 years (*n* =110)	1,667 (1,570, 1,763)	520 (459, 581) (31%)	594 (548, 640) (36%)	389 (344, 434) (23%)	140 (117, 163) (8%)	16 (9,24) (1%)	7 (1,12) (<1%)
BMI categories *	Normal (*n* =170)	1,679 (1,599, 1,759)	602 (546, 657) (36%)	563 (514, 612) (34%)	332 (299, 366) (20%)	168 (144, 192) (10%)	10 (5,15) (1%)	4 (2,6) (<1%)
	Overweight (*n* =37)	1,555 (1,387, 1,722)	517 (425, 610) (33%)	535 (454, 616) (34%)	329 (246, 411) (21%)	159 (108, 210) (10%)	4 (<1, 7) (<1%)	11 (-2, 24) (1%)
	Obese (*n* =61)	1,513 (1,383, 1,643)	461 (377, 546) (30%)	529 (460, 598) (35%)	351 (277, 424) (23%)	160 (130, 190) (11%)	9 (1,16) (1%)	3 (1,5) (<1%)
Main meals	Breakfast (*n* = 235) ^∧^	297 (234, 376)	114 (99, 128) (38%)	73 (61, 86) (25%)	47 (19, 75) (16%)	57 (37, 77) (19%)	2 (-0.1, 5) (1%)	4 (-1, 10) (1%)
	Lunch (*n* =275) ^∧^	451 (407, 470)	103 (91, 115) (23%)	165 (145, 185) (37%)	87 (76, 99) (19%)	83 (69, 97) (18%)	2 (1,3) (2%)	11 (5,17) (11%)
	Dinner (*n* =284)	533 (498, 555)	100 (91, 115) (19%)	179 (162, 196) (34%)	170 (152, 188) (32%)	66 (56,76) (12%)	8 (4,13) (2%)	9 (5,13) (2%)
Snacks^∧^	Morning snack (*n* =266)	338 (243, 395)	106 (92, 121) (31%)	77 (65,89) (23%)	70 (53, 87) (21%)	55 (45, 64) (16%)	8 (<1, 16) (2%)	22 (-8, 52) (6%)
	Afternoon snack (*n* =273)	370 (290, 396)	99 (87, 112) (27%)	91 (72, 111) (25%)	90 (80, 117) (27%)	57 (48, 66) (15%)	4 (1,6) (1%)	20 (-0.3, 41) (5%)
	Evening snack (*n* =252)	366 (286, 376)	93 (79, 106) (25%)	76 (52, 99) (21%)	123 (101, 146) (34%)	55 (40, 71) (21%)	11 (1,21) (3%)	8 (1,16) (2%)
	Late evening snack (*n* =130)	274 (242, 298)	68 (48,89) (25%)	39 (22, 55) (14%)	89 (51, 127) (33%)	56 (18, 95) (21%)	9 (<1, 20) (3%)	13 (-82, 107) (5%)

* Indicates missing data for 16 participants; ∧ Not all adolescents had consumption during the stated meals.

There was no statistically significant difference in the percentage of energy from each taste between boys and girls. However, younger adolescents (10–14 years) had higher energy intake from sweet-tasting foods by 6% (95%CI 1, 9; *P* < 0.01), and higher salty-tasting foods by 3% (95%CI 1, 5; *P* < 0.01) compared with older adolescents (15–19 years). Older adolescents had significantly higher energy intakes from savory-tasting foods by 5% (95%CI 2, 7; *P* < 0.01), and bitter-tasting foods by 1% (95%CI 0.2, 1; *P* < 0.01) compared to younger individuals. In relation to BMI categories, normal-weight individuals had a borderline significant difference in the energy intake from sweet-tasting foods compared to obese individuals by 6% (95%CI 0.03, 10; *P* = 0.05).

At breakfast, most of the energy intake was obtained from sweet-tasting foods (38%) while energy intakes from neutral-tasting foods were higher at lunch (37%) and dinner (34%). Across main meals, adolescents had significantly higher energy intake from sweet-tasting foods at breakfast compared to lunch-time by 15% (95%CI 7, 19; *P* < 0.01) and at dinner by 19% (95%CI 11, 23; *P* < 0.01). Energy intake from savory-tasting foods at dinner was higher by 13% (95%CI 6, 18; *P* < 0.01) than at lunch. Energy intake from neutral-tasting foods at lunch and dinner was significantly higher than at breakfast.

For snacks, adolescents had a higher energy intake from sweet-tasting foods in the morning (31%). In the afternoon, sweet-tasting foods and savory-tasting foods were the major contributors to the energy intake by 27% each. Savory-tasting foods were also the main source of energy intake for snacking in the evening (34%) and for late snacking (33%). However, no significant differences in energy intake were observed across the different snacking times.

### Assessment of the UK Adolescents' Dietary Taste Based on Their Food Records From the NDNS

Table 2 illustrates the nutrient and food intake by taste. Taste is characterized as a percentage of the total food weight presented by quintile.

**Table 2:** Characteristics of adolescents' dietary intakes by the quintiles (Q) weight of foods consumed as a percentage of the total food weight.

	**Quintiles of sweet-tasting foods as percentage of the total food weight** (%)							
	**Q1 (*n* = 57)**	**Q2 (*n* = 57)**	**Q3 (*n* = 57)**	**Q4 (*n* = 57)**	**Q5 (*n* = 56)**	**%Diff Q1&Q5**	Coeff. (95% CI)^*^	***P*** **-trend**
	**7–31%**	**31–37%**	**37–43%**	**43–50%**	**50-73%**			
Energy (kcal/d)	1,449 (1,330, 1,569)	1,574 (1,428, 1,721)	1,696 (1,564, 1,828)	1,750 (1,620, 1,879)	1,738 (1,619, 1,858)	20%	10 (6, 15)	<0.01
Carbohydrate (g/d)	183 (169, 198)	208 (186, 231)	223 (209, 237)	234 (216, 253)	250 (235, 266)	37%	2 (1.5, 3)	<0.01
Protein (g/d)	62 (56, 67)	62 (56, 68)	65 (59, 72)	69 (6276)	58 (53, 64)	−6%	0.02 (−0.2, 0.2)	0.83
Fat (g/d)	57 (51, 63)	60 (54, 66)	66 (59, 74)	65 (59, 71)	62 (56, 68)	9%	0.3 (0.03, 0.5)	0.02
Total sugars (g/d)	54 (48, 61)	71 (62, 80)	89 (80, 99)	96 (87, 105)	116 (105, 127)	115%	2 (1.5, 2)	<0.01
Free sugars (g/d)	34 (28, 40)	47 (40, 54)	61 (51, 72)	63 (51, 74)	84 (72, 97)	147%	1.5 (1, 2)	<0.01
Fibre (g/d)	14 (13, 15)	16 (14, 18)	15 (13, 16)	16 (14, 17)	15 (14, 17)	7%	0.04 (−0.01, 0.1)	0.14
Saturated fat (g/d)	19 (17, 21)	22 (19, 25)	24 (21, 28)	26 (23, 28)	25 (22, 28)	32%	0.2 (0.1, 0.3)	<0.01
Sodium (mg/d)	1,791 (1,580, 2,003)	1,772 (1,584, 1,961)	1,983 (1,771, 2,195)	1,942 (1,769, 2,114)	1,651 (1,456, 1,846)	−8%	1 (−7, 8)	0.86
Fruit (g/d)	55 (37, 73)	52 (33, 72)	69 (50, 88)	71 (42, 100)	88 (58, 118)	60%	1.2 (0.3, 2)	<0.01
Fruit juice (g/d)	57 (25, 89)	72 (32, 112)	88 (60, 117)	82 (44, 120)	149 (73, 225)	161%	2 (0.3, 4)	0.02
Brassica vegetables (g/d)	12 (6, 19)	12 (7, 17)	16 (7, 24)	10 (4, 16)	10 (5, 15)	−17%	−0.04 (−0.2, 0.2)	0.66
Other vegetables (g/d)	87 (69, 105)	97 (77, 117)	73 (62, 84)	106 (83, 130)	73 (57, 90)	−16%	−0.3 (−1, 0.4)	0.42
Meat & poultry (g/d)	72 (56, 89)	55 (44, 65)	72 (51, 93)	59 (47, 72)	45 (35, 55)	−38%	−0.5 (−1, −0.3)	0.03
Processed meats (g/d)	25 (17, 33)	26 (17, 35)	29 (21, 36)	28 (19, 36)	18 (11, 26)	−28%	−0.1 (−0.4, 0.3)	0.66
Cheese (g/d)	18 (12, 24)	22 (15, 28)	16 (11, 22)	17(12, 23)	18 (13, 23)	0%	−0.1 (−0.3, 0.1)	0.55
	**Quintiles of neutral-tasting foods as percentage of the total food weight (%)**							
	**Q1 (*****n** =* **57)**	**Q2 (*****n** =* **57)**	**Q3 (*****n** =* **57)**	**Q4 (*****n** =* **57)**	**Q5 (*****n** =* **56)**	**%Diff Q1&Q5**	**Coeff. (95% CI)** ^*^	***P-*** **trend**
	**9–26%**	**26–33%**	**33–38%**	**38–46%**	**46–78%**			
Energy (kcal/d)	1,772 (1,647, 1,898)	1,721 (1,580, 1,863)	1,644 (1,537, 1,751)	1,601 (1,445, 1,757)	1,436 (1,317, 1,555)	−19%	−10 (−15, −5)	<0.01
Carbohydrate (g/d)	243 (225, 261)	228 (210, 247)	218 (204, 233)	211 (190, 233)	191 (173, 209)	−21%	−2 (−2, −1)	<0.01
Protein (g/d)	60 (54, 65)	69 (62, 76)	66 (60, 72)	63 (56, 70)	58 (53, 63)	−3%	−0.1 (−0.4, 0.1)	0.25
Fat (g/d)	69 (61, 76)	64 (56, 71)	61 (57, 66)	62 (55, 69)	55 (49, 60)	−20%	−0.4 (−1, −0.1)	0.02
Total sugars (g/d)	111 (98, 124)	93 (83, 103)	80 (72, 87)	77 (66, 87)	59 (52, 67)	−47%	−1 (−2, −1)	<0.01
Free sugars (g/d)	82 (69, 95)	63 (52, 74)	52 (44, 59)	51 (41, 60)	38 (31, 44)	−54%	−1 (−2, −1)	<0.01
Fibre (g/d)	15 (13, 16)	16 (14, 17)	16 (14, 17)	15 (13, 17)	14 (13, 16)	−7%	−0.02 (−0.1,0.03)	0.48
Saturated fat (g/d)	28 (24, 32)	25 (21, 28)	22 (21, 24)	22 (19, 25)	18 (16, 20)	−36%	−0.3 (−0.4, −0.2)	<0.01
Sodium (mg/d)	1,845 (1,605, 2,085)	2,013 (1,848, 2178)	1,841 (1,611, 2,072)	1,801 (1,604, 1,997)	1,648 (1,473, 1,823)	−11%	−7 (−15, 1)	0.07
Fruit (g/d)	64 (39, 89)	72 (50, 93)	68 (40, 95)	64 (45, 84)	60 (42, 79)	−6%	−0.1 (−1, 1)	0.73
Fruit juice (g/d)	131 (56, 206)	90 (44, 137)	86 (53, 118)	67 (34, 100)	66 (32, 101)	−50%	−1 (−4, 1)	0.19
Brassica vegetables (g/d)	10 (5, 15)	13 (7, 20)	12 (5, 20)	12 (6, 17)	12 (6, 19)	20%	0.01 (−0.2, 0.2)	0.94
Other vegetables (g/d)	77 (60, 94)	106 (80, 132)	84 (73, 95)	82 (64, 100)	89 (69, 108)	16%	−0.1 (−1, 1)	0.81
Meat and poultry (g/d)	44 (34, 53)	63 (51, 75)	66 (45, 86)	70 (53, 86)	62 (49, 76)	41%	0.4 (−0.1, 1)	0.13
Processed meats (g/d)	32 (22, 43)	32 (24, 40)	20 (14, 26)	25 (17, 32)	18 (11, 25)	−44%	−0.4 (−1, −0.1)	<0.01
Cheese (g/d)	23 (16, 30)	18 (13, 23)	20 (13, 26)	17 (12, 22)	16 (10, 21)	−30%	−0.2 (−0.4, 0.03)	0.10
	**Quintiles of savory-tasting foods as percentage of the total food weight (%)**							
	**Q1 (*****n** =* **57)**	**Q2 (*****n** =* **57)**	**Q3 (*****n** =* **57)**	**Q4 (*****n** =* **57)**	**Q5 (*****n** =* **56)**	**%Diff Q1&Q5**	**Coeff. (95% CI)** ^*^	***P*** **-trend**
	**0–7%**	**7–10%**	**10–12%**	**12–16%**	**16–27%**			
Energy (kcal/d)	1,678 (1,565, 1,791)	1,698 (1,566, 1,831)	1,609 (1,476, 1,741)	1584 (1,443, 1,725)	1,581 (1,433, 1,730)	−6%	−9 (−19, 0.4)	0.06
Carbohydrate (g/d)	233 (216, 249)	231 (213, 249)	220 (201, 240)	207 (188, 227)	200 (181, 220)	−14%	−3 (−4, −1)	<0.01
Protein (g/d)	58 (53, 62)	63 (58, 69)	61 (56, 66)	65 (58, 72)	66 (59, 73)	14%	0.4 (−0.01, 1)	0.05
Fat (g/d)	63 (57, 69)	64 (57, 70)	60 (53, 66)	60 (54, 66)	63 (55, 70)	0%	−0.2 (−1, 1)	0.92
Total sugars (g/d)	100 (88, 113)	89 (78, 100)	89 (76, 102)	71 (62, 80)	70 (61, 79)	−30%	−2 (−3, −1)	<0.01
Free sugars (g/d)	69 (57, 82)	59 (50, 69)	65 (52, 77)	45 (37, 53)	47 (37, 57)	−32%	−2 (−2, −1)	<0.01
Fibre (g/d)	16 (14, 17)	16 (15, 18)	13 (12, 15)	15 (14, 17)	14 (12, 16)	−13%	−0.1 (−0.2, 0.01)	0.07
Saturated fat (g/d)	25 (21, 28)	24 (21, 26)	23 (20, 25)	21 (19, 24)	22 (19, 26)	−12%	−0.1 (−0.4, 0.2)	0.42
Sodium (mg/d)	1,762 (1,637, 1,888)	1,810 (1,639, 1,981)	1,734 (1,553, 1,915)	1,925 (1,690, 2,160)	1,840 (1,600, 2,080)	4%	7 (−9, 23)	0.37
Fruit (g/d)	100 (70, 129)	85 (61, 109)	50 (33, 66)	48 (35, 60)	53 (30, 75)	−47%	−3 (−5, −1)	<0.01
Fruit juice (g/d)	97 (53, 140)	133 (65, 202)	77 (50, 104)	53 (33, 73)	84 (31, 136)	−13%	−3 (−7, 1)	0.12
Brassica vegetables (g/d)	7 (3, 11)	17 (8, 25)	8 (3, 12)	13 (6, 21)	13 (8, 19)	86%	0.2 (−0.2, 1)	0.39
Other vegetables (g/d)	65 (51, 80)	91 (69, 112)	82 (63, 101)	97 (81, 113)	97 (76, 118)	49%	2 (0.2, 3)	0.03
Meat & poultry (g/d)	38 (29, 46)	59 (48, 71)	60 (48, 71)	71 (54, 88)	72 (54, 90)	90%	2 (0.4, 3)	0.01
Processed meats (g/d)	21 (14, 27)	26 (17, 35)	20 (14, 26)	22 (15, 29)	35 (25, 44)	67%	1 (0.2, 2)	0.01
Cheese (g/d)	18 (13, 23)	21 (15, 27)	19 (15, 24)	15 (9, 20)	20 (13, 27)	11%	0.1 (−1, 1)	0.85
	**Quintiles of salty-tasting foods as percentage of the total food weight (%)**							
	**Q1 (*****n** =* **57)**	**Q2 (*****n** =* **57)**	**Q3 (*****n** =* **57)**	**Q4 (*****n** =* **57)**	**Q5 (*****n** =* **56)**	**%Diff Q1&Q5**	**Coeff. (95%CI)** ^*^	***P*** **-trend**
	**0–3%**	**3–6%**	**6–8%**	**8–11%**	**11–31%**			
Energy (kcal/d)	1,617 (1,474, 1,760)	1,655 (1,493, 1,816)	1,579 (1,439, 1,720)	1,649 (1,536, 1,762)	1,621 (1,499, 1,742)	0%	2 (−9, 12)	0.72
Carbohydrate (g/d)	214 (195, 234)	226 (201, 250)	210 (194, 227)	226 (209, 244)	206 (191, 220)	−4%	−0.4 (−2, 1)	0.50
Protein (g/d)	67 (59, 74)	66 (60, 73)	61 (55, 67)	59 (54, 63)	63 (57, 68)	−6%	−0.3 (−1,.2)	0.26
Fat (g/d)	60 (54, 67)	61 (54, 67)	60 (52, 68)	62 (57, 68)	66 (59, 73)	10%	1 (−0.1, 1)	0.07
Total sugars (g/d)	83 (72, 94)	84 (72, 96)	87 (74, 100)	89 (77, 101)	69 (61, 77)	−17%	−0.7 (−2, 0.1)	0.08
Free sugars (g/d)	53 (42, 64)	53 (42, 63)	63 (51, 75)	64 (52, 75)	47 (40, 54)	−11%	−0.2 (−1, 1)	0.65
Fibre (g/d)	15 (14, 17)	15 (13, 17)	14 (13, 16)	15 (13, 16)	15 (14, 17)	0%	−0.02 (−0.1, 0.1)	0.80
Saturated fat (g/d)	22 (19, 25)	22 (19, 25)	22 (19, 26)	23 (21, 25)	25 (22, 29)	14%	0.3 (0.02, 1)	0.03
Sodium (mg/d)	1,770 (1,545, 1,996)	1,717 (1,511, 1,923)	1,711 (1,523, 1,898)	1,825 (1,642, 2,008)	2,101 (1,893, 2,309)	19%	22 (4.5, 40)	0.01
Fruit (g/d)	66 (49, 83)	92 (62, 122)	64 (43, 85)	66 (44, 89)	35 (23, 47)	−47%	−2 (−4, −1)	<0.01
Fruit Juice (g/d)	70 (35, 104)	66 (35, 98)	111 (50, 172)	126 (67, 184)	60 (31, 89)	−14%	−0.5 (−3, 2)	0.73
Brassica vegetables (g/d)	19 (10, 28)	11 (6, 15)	12 (6, 19)	10 (6, 15)	7 (3, 11)	−63%	−1 (−1, −0.2)	<0.01
Other vegetables (g/d)	101 (81, 120)	91 (71, 111)	91 (74, 107)	78 (56, 100)	77 (62, 92)	−24%	−2 (−3, −0.3)	0.01
Meat & poultry (g/d)	75 (53, 96)	72 (57, 87)	56 (46, 66)	57 (47, 67)	42 (33, 52)	−44%	−2 (−3, −1)	<0.01
Processed meats (g/d)	14 (8, 21)	22 (14, 31)	22 (15, 28)	28 (22, 35)	40 (30, 49)	186%	2 (1, 2)	<0.01
Cheese (g/d)	10 (6, 14)	15 (10, 19)	18 (14, 22)	19 (14, 24)	33 (26, 41)	230%	1 (1, 2)	<0.01
	**Quintiles of bitter-tasting foods as percentage of the total food weight (%)**							
	**Q1 (*****n** =* **88)**	**Q2 (*****n** =* **26)**	**Q3 (*****n** =* **57)**	**Q4 (*****n** =* **57)**	**Q5 (*****n** =* **56)**	**%Diff Q1&Q5**	**Coeff. (95%CI)** ^*^	***P-*** **trend**
	**0%**	<**1–1%**	**1–4%**	**4–7%**	**7–27%**			
Energy (kcal/d)	1,570 (1,454, 1,686)	1,808 (1,584, 2,032)	1,607 (1,501, 1,713)	1,673 (1,517, 1,828)	1,585 (1,475, 1,696)	1%	−3 (−15, 9)	0.62
Carbohydrate (g/d)	213 (196, 231)	245 (211, 278)	219 (207, 231)	215 (195, 235)	210 (192, 228)	−1%	−1 (−3, 1)	0.30
Protein (g/d)	58 (54, 62)	73 (62, 84)	60 (53, 67)	67 (61, 74)	63 (57, 69)	9%	0.2 (−0.4, 1)	0.41
Fat (g/d)	60 (55, 65)	67 (59, 75)	61 (55, 66)	66 (58, 74)	58 (54, 63)	−3%	−0.3 (−1, 0.2)	0.28
Total sugars (g/d)	81 (70, 93)	91 (75, 107)	86 (76, 97)	78 (67, 89)	82 (72, 93)	1%	−0.2 (−1, 1)	0.77
Free sugars (g/d)	57 (47, 67)	57 (44, 71)	58 (47, 69)	54 (44, 64)	54 (43, 65)	−5%	−0.3 (−1, 1)	0.59
Fibre (g/d)	14 (13, 15)	18 (15, 21)	15 (14, 16)	15 (14, 17)	15 (13, 16)	7%	−0.02 (−0.2, 0.2)	0.86
Saturated fat (g/d)	23 (20, 25)	26 (22, 30)	21 (20, 23)	25 (21, 28)	21 (19, 23)	−9%	−0.2 (−0.4, 0.1)	0.15
Sodium (mg/d)	1,722 (1,590, 1,855)	2,036 (1,763, 2,309)	1,779 (1,535, 2,023)	1,906 (1,660, 2,152)	1,803 (1,664, 1,941)	5%	1 (−15, 18)	0.86
Fruit (g/d)	69 (50, 89)	85 (38, 132)	72 (49, 96)	54 (36, 72)	59 (39, 79)	−14%	−1 (−4, 1)	0.34
Fruit juice (g/d)	86 (56, 115)	118 (60, 176)	120 (50, 190)	69 (41, 97)	61 (21, 102)	−29%	−4 (−8, 1)	0.11
Brassica vegetables (g/d)	6 (2, 10)	8 (2, 13)	17 (10, 25)	12 (6, 18)	15 (9, 21)	150%	1 (−0.1, 1)	0.07
Other vegetables (g/d)	63 (51, 74)	112 (73, 151)	97 (78, 115)	84 (68, 100)	104 (85, 124)	65%	3 (1, 5)	0.01
Meat & poultry (g/d)	53 (42, 65)	55 (37, 73)	64 (45, 84)	65 (53, 76)	66 (51, 81)	25%	1 (−1, 2)	0.41
Processed meats (g/d)	21 (16, 26)	43 (28, 58)	20 (14, 26)	30 (21, 39)	22 (16, 29)	5%	−0.1 (−1, 1)	0.81
Cheese (g/d)	17 (13, 22)	23 (15, 32)	15 (9, 20)	25 (18, 31)	15 (11, 19)	−12%	−0.2 (−1, 0.3)	0.51

* Change in nutrient/food per % increase in taste.

Q1–Q5 = quintiles 1 (lowest quintile)- quintiles 5 (highest quintile). Each quintile represents: (1) number of adolescents (n); although they are in the same size it is different individuals; (2) proportion of food tastes (%).

### Sweet-Tasting Foods

Energy, carbohydrate, sugars, and saturated fat all showed significant positive linear trends with increasing sweet-tasting foods. Energy intake increased by 20% from the lowest quintile (Q1) to the highest quintile (Q5) and there was a statistically significant positive trend of higher energy intake by 10 kcal/d (95% CI 6, 15; *P* < 0.01) for each percentage increase in sweet food consumption. Carbohydrate intake also showed a positive overall trend of higher intakes with higher sweet foods. Individuals who had the highest proportion of sweet-tasting foods (Q5) had higher total sugar (115%) and free sugar (147%) intakes compared to those in the lowest quintile (Q1). Total fat intake was 9% higher between the lowest quintile (Q1) to the highest quintile (Q5) of sweet-tasting foods with an overall significant trend (*P* = 0.02).

Fruit intake was 60% higher and fruit juice was 161% higher in the highest quintile (Q5) compared to the lowest quintile (Q1) of sweet-tasting foods with overall significant trends for both. Meat and poultry intakes were 38% lower between the lowest and highest quintile (Q5) with an overall significant trend (*P* = 0.03).

### Neutral-Tasting Foods

Energy, carbohydrate, sugars, total fat and saturated fats all showed significant negative linear trends with increasing neutral-tasting foods. Energy intake decreased by 19% from the lowest to the highest quintile and there was a statistically significant negative trend of lower energy intake by 10 kcal/d (95% CI −15, −5; *P* < 0.001) for each increase in the proportion of neutral-tasting foods. Individuals in the highest quintile of neutral-tasting foods had lower carbohydrate (21%), total sugars (47%), and free sugars (54%) compared to those in the lowest quintile. Total fat and saturated fats intakes also showed negative overall trends of lower intakes with higher consumption of neutral-tasting foods. Processed meats consumption was 44% lower in the highest compared to the lowest quintile of neutral-tasting foods; with an overall significant trend (*P* < 0.01) per each percentage increase in neutral-tasting foods.

### Savory-Tasting Foods

Protein intake showed a borderline significant positive linear trend while carbohydrate and sugars intakes showed inverse linear trends with higher consumption of savory-tasting foods. Individuals in the highest quintile of savory-tasting foods had 14% higher protein intake compared with those in the lowest quintile. Carbohydrate intake decreased by 14% from the lowest to the highest quintiles. Also, total sugars intake was (30%) lower and free sugars intake was (32%) lower between the lowest and highest quintiles.

Fruit intake was inversely associated with higher amounts of savory-tasting foods; with a 47% lower intake between the highest and lowest quintile and overall decrease per each percentage increase in savory foods by 3 g/d (95% CI −5, −1; *P* < 0.001). However, non-Brassica vegetable intake was higher with increasing amounts of savory foods. Meat and poultry intakes increased by 90% from the lowest to the highest quintile and there was a statistically significant positive trend of higher meat intake by 2 g/d (95% CI 0.4, 3; *P* = 0.01) with each percentage increase in savory-tasting foods. Processed meats intake increased by 67% from the lowest to the highest quintile.

### Salty-Tasting Foods

Individuals with the lowest proportion of salty foods (Q1) had 19% less sodium, 1,771 mg/d (95% CI 1,545, 1,996) compared to individuals with the highest proportion of salty foods (Q5) 2,101 mg/d (95% CI 1,893, 2,309). Overall sodium intake was higher by 22 mg/d (95% CI 5, 40; *P* = 0.01) for each percentage increase in salty foods. Saturated fats intake increased by 14% from the lowest quintile (Q1) to the highest quintile (Q5) and there was a statistically significant positive trend of higher intake by 0.3 g/d (95% CI 0.02, 1.00; *P* = 0.03) for each percentage increase in salty foods. Processed meat consumption was 186% higher and cheese intake 230% higher between the lowest to the highest quintile. Non-processed meat and poultry showed an overall negative trend of 2 g/d (95% CI 3, 1; *P* = 0.02) lower for each percentage increase in salty foods consumed. Similarly, higher intakes of both fruit and Brassica vegetables were associated with lower intakes of salty foods.

### Bitter-Tasting Foods

The proportion of bitter-tasting foods was not shown to have a statistically significant association with dietary intakes, except with vegetables. The intake of non-Brassica vegetables increased by 65% from the lowest to the highest quintile and there was a significant positive trend of higher non-Brassica vegetable intake by 3 g/d (95% CI 1, 5; *P* < 0.01) with each percentage increase in bitter-tasting foods. Brassica vegetables also increased by 150% from the lowest quintile (Q1) to the highest quintile (Q5); with a borderline significant positive trend of higher Brassica vegetables intake by 1 g/d per percentage increase in bitter foods (95% CI −0.1, 1.0; *P* = 0.07).

### Sour-Tasting Foods

As seen in Supplementary Table S6, only 70 adolescents (25%) had any intake from sour-tasting foods. There was no statistically significant association between any of the nutrients explored and the sour-tasting foods. Individuals who consumed sour-tasting foods had higher intakes of Brassica vegetable 16 g/d (95% CI 10, 23) compared with non-consumers 10 g/d (95% CI 7, 13) and there was a significantly higher intake by 2 g/d (95% CI 0.5, 4; *P* = 0.01) for each percentage increase in sour foods. Meat & poultry intakes were also higher among consumers of sour-tasting foods.

## Discussion

The present study aimed to characterize the taste of UK adolescents' overall food and nutrient intakes using food records from the UK National Diet and Nutrition Survey, NDNS (2016–2017). Our approach of characterizing the food taste of the whole diet is novel in this age group. Findings revealed that taste contributions to daily energy intake differed based on sample characteristics and eating occasions. Findings have also shown different trends in the intake of nutrients and foods according to the contribution of each taste to the overall diet.

Comparing the taste classification from our work against previous published work using trained panelists showed a good level of agreement for foods which were available; suggesting that taste classification by regular consumers could be reliable. The small number of disagreements between our survey and trained panelists may be due to a range of factors including variations in ingredients, food preparation and other factors that could affect the taste of the crops including ripeness, seasonality, and different types of tested items (e.g., there are sweet tomatoes, while others are sour, savory or neutral).

About two-thirds of adolescents' dietary intakes were from both sweet-tasting and neutral-tasting foods, and around one third were from both savory and salty-tasting foods. However, taste contributions to daily energy intake differed by age group. Young individuals have been shown to have greater preference and consumption of sweet-tasting foods than adults (38, 39). Adults may consume more bitter-tasting foods due to their awareness of potential health benefits (36). This may explain our findings of higher energy intake from sweet foods among younger adolescents compared with older adolescents whose highest energy intake was from neutral-tasting foods. Also, older adolescents were observed to have a higher energy intake from bitter-tasting foods compared with younger individuals. This was linked to higher consumption of coffee, tea, and alcoholic beverages where the bitterness in those items was found to be acceptable (45). Concerning savory and salty foods, older adolescents had slightly higher energy intake from these tastes compared to younger adolescents. A study on adolescents' frequent consumption of takeaway foods at age 12 and followed-up at age 17 found increasing consumption by age (46). Takeaway foods alongside other items (e.g., crisps and nuts), were classified as salty or savory tastes in the current work.

Sweet-tasting foods dominated breakfast-times, which may be due to the intake of milk, breakfast cereals, white bread, sugar preserves, sweet spreads, and/or fruit which have been reported as popular foods consumed by the UK population at breakfast (47). Sweet tasting foods also contributed the most energy for daytime snacking. An earlier study comparing adolescents snacking showed that sugar-sweetened beverages, caloric-dense foods (e.g., biscuits, cakes, and pastries), and fruit were the most commonly consumed snacks (48). However, we found that later on the day, at lunch and dinner as well as evening and late evening snacking, foods tasting neutral, and savory were the highest sources of the adolescents' energy intake. This could be explained by the common consumption of core foods at lunch and dinner (composite dishes like meat and poultry-based foods and some vegetables) and savory snacks.

Evidence on the relationship between BMI and taste is contradictory. Studies on adults have shown a positive association between higher BMI and preference for savory and salty foods (22, 49, 50) and sweet foods (49, 50), while others observed no association (51). Normal-weight adults have reported preferring sweet foods more than adults with obesity (22). A study characterizing adolescent tastes by genotype observed a higher intake of chocolate among individuals with obesity than normal weight (52) while in another study, a higher preference for salty foods was reported by overweight and obese adolescents (53). In our study, normal-weight individuals had higher energy intake from sweet foods compared with those with obesity who had the highest energy intake from neutral-tasting foods and both of savory and salty tasting foods. A similar result has also been shown in adults (26). However, inconsistent findings may be attributed to a number of possible reasons. First, the methods used in assessing taste are varied which may influence the outcome (16, 54). Second, whether bodyweight is measured or self-reported may have an effect. Differences between self-reported and measured body weight were associated with differences in taste perception (55). Third, potential misreporting of certain foods in food records may affect the outcome association between taste and BMI (56, 57). Fourth, the relationship between taste and body weight may depend on age and gender. Older individuals and girls identified tastes better than younger individuals and boys (55, 58). Fifth, leptin, which is associated with higher body weight, has been found to decrease sweetness perception which could drive individuals to consume higher concentrated sweet taste foods. This could affect the taste buds causing taste impairment associated with obesity (59). Furthermore, tastes, and contributing components such as sugar and salt increase food palatability and hedonic responses that could be linked with increased consumption (60). This could cause potential health risks, especially with the presence of obesity.

Regarding the overall characteristic of adolescents' dietary intake by taste, we found that higher consumption of sweet-tasting foods was linked to a higher intake of energy, carbohydrate, sugars, fiber, and saturated fats. Previous studies have also identified a strong association between sweetness and sugar content in foods (23–25), and liking for higher concentrations of sweet taste was positively associated with total energy, carbohydrate and total sugar intake in adults (61). Adolescents' eating is often categorized by high calorie-dense food with a high proportion of calories coming from fat and sugar (28, 62, 63). It has been reported that children and adolescents have the highest intake of free sugars; at least three times the recommended level. This high consumption of added sugars has been a public health concern due to the potential of free sugars increasing the risk of obesity and consequently other non-communicable diseases (64). In our study, we observed adolescents' intake of free sugars exceeded the dietary recommendations of < 5% (64). This could be explained by the consumption of sweet snacks and sweet baked products which highly contributed to the sweet taste in the present study. This was the opposite of the observations from adults who had low consumption of sweet-tasting foods and drinks and sucrose intake associated with increased intensity of the sweetness (61). However, another study on adults reported higher intake of sweetened beverages and high energy intake from sugar-sweetened beverages among those who reported higher preference for sweetness compared with others who showed less or neutral liking (65). In the current study, it was noted by the food records that adolescents had frequent consumption of sweet beverages, especially, with meals. A review has reported that approximately 75% of calorie-dense beverage consumption (e.g., carbonated soft drinks) occurs with meals (66). Moreover, the addition of sugar to coffee and tea could be contributed to the high level of sugar intake where sweetness modulates the acceptance of the bitterness of these beverages (67). Nevertheless, as sweet taste is related to the calorie content in food and energy intake, the increased consumption of sweet-tasting foods among adolescents may indicate the increased need for calories during this period of growth (68). However, healthier choices of sweet foods and beverages are recommended.

Adolescents had a higher protein intake associated with a higher intake of savory-tasting foods compared with the other tastes. This may be related to the higher consumption of meats and processed meats. Previous work has reported moderate (23) to strong correlation between savory-tasting foods and protein content (25). Protein and sodium contents were found to have positive associations with saltiness (25). Whilst studies on adults reported that individuals with higher preference for salty taste had a higher intake of fast-foods, protein (69) and protein-source foods (e.g., legumes, and white meats) (70), our findings confirmed the positive association between sodium intake and the higher intake of salty-tasting foods, but protein intake did not increase with saltiness. This could be explained by the observed higher intake of sodium sources (e.g., cheese and processed meats) and the lower intake of protein sources (e.g., meat and poultry) within the higher intake of salty-tasting foods. Whilst these findings indicate a link between sodium intake and saltiness (24, 25); this has not always been found to be true (23). Interestingly, processed meats were found to correlate with both savory and salty tastes, which may refer to a potential connection between these tastes. This is because processed products (e.g., some type of cheese and processed meats) are high in salt and other taste enhancing items including monosodium glutamate (MSG). MSG is known for its savory taste, which can also enhance the saltiness in the foods (71–73). However, some foods (e.g., meats, mushroom) also naturally produce savory taste due to the presence of the amino aide, glutamate (74). Regarding the findings in relation to neutral-tasting foods, the high consumption was negatively associated with the intake of energy and most of the nutrients. This could be due to the relatively low taste intensity in the foods classified as neutral (75) which failed to demonstrate taste-nutrient relationships.

The UK dietary guidelines recommend at least five portions of fruit and vegetables a day (76). Adolescents' intake of fruit and vegetables has been reported to be low (33). The Health Survey for England (HSE) found that young adults (aged 16 to 24 years) did not meet the recommendation of fruit and vegetable portion size; and that <18% of UK children aged 5–15 years ate five portions of fruits and vegetables (77). Our findings showed that a higher intake of fruit was associated with the higher intake from sweet-tasting foods, while a higher vegetable intake was observed with higher intakes of bitter-, savory- and neutral-tasting foods; although, the guideline of five-a-day was typically not met (about 3 portions of fruits and vegetables were consumed/day). The current results indicated a positive association between vegetable consumption and bitter taste. In our earlier systematic review, we reported findings from genotype and phenotype studies on adolescents linking to bitter taste. Perceived bitterness was negatively associated with the preference of foods with bitter taste including Brassica vegetables (12). Likewise lower intakes of coffee (17, 21), beer, and Brussel sprouts have been observed in highly bitter sensitive adults compared with those who are less sensitive (17); yet, this is not always true in adults due to cognitive control. However, adolescents were found to eat vegetables as part of composite foods, and rarely consumed vegetables on their own (78, 79). There may be a role of saltiness (80–82), savory/umami taste (81, 83), and fats (84, 85) in meals which suppress the bitterness. This may explain our results of higher consumption of meats, and vegetables among adolescents in association with the higher intake of bitter-and savory-tasting foods.

To our knowledge, this is the first study to assess adolescents' dietary intakes from a taste perspective using nationally representative food intake data. While our approach of using regular consumers was subjective, this is true for all phenotype methods used in sensory studies. Moreover, there is no universally agreed or standard method to assess taste patterns, but we still found agreement with other studies. Additionally, the significant associations between taste (e.g., higher intake of sweet, salty or savory tasting foods) and nutrient intake (e.g., intake of sugar, sodium or protein, respectively) that we observed agrees with previous work using trained panelists (23–25). However, some limitations are worth mentioning. The first limitation is related to the food diary method that was used for collecting the dietary data, which is subject to potential recording bias, omission of foods and misreporting of some foods or portion sizes. Altering dietary behaviors is also a potential problem as a result of a lack of motivation, the burden of recording or to demonstrate good dietary habits. Moreover, under-reporting is expected with dietary measurements, especially with multiple recording days (86), which potentially has an impact on estimations of food and nutrient intakes (87). Additionally, under-reporting some foods could have affected the taste classification of foods, proportion of foods in taste groups and taste contribution to energy intake and its influence on dietary intake in general. Another limitation is our use of adults to characterize food tastes which was due to the COVID 19 situation and ethical constraints which limited us approaching adolescents. Although we considered including varieties of representative foods to be matched to the entire foods in the NDNS, some variations in taste and/or intensity may differ due to the use of different ingredients, herbs, or spices. This leads to another limitation that our approach of classifying the main taste for each food may not take the taste profile of food and tastes interactions into consideration. We only used 1 year of adolescents' data from the NDNS. Although we applied the sampling weight in the analysis for a more representative set of results, it may not be suitable to generalize the findings. Future work could use additional years of adolescents' data from the UK national survey, which would also allow a wider range of foods to be classified by taste.

The current work characterized adolescents' food intake by taste as a first step in understanding the effect of taste on this age group's dietary intake. However, since foods are often eaten in combination involving different tastes, it would be more valuable to study the role of taste on their dietary patterns by exploring their dietary taste patterns. Also, while taste may have an influence on the diet quality, a limited number of studies have explored that and the studies are limited to specific tastes (88, 89). Furthermore, others only reported that participants who rated taste as a very important factor had poor diet quality, although they did not study the association between dietary taste and diet quality directly (3, 90). In a recent study, authors have reported poor diet quality associated with sweet foods other than fruit (e.g., ice cream, biscuits, chocolate, sweetened beverages) and salty foods (e.g., crisps, chips, fast foods) (91). In contrast, a study by Cox and colleagues reported good diet quality associated with sweet and bitter foods but not salty foods (92). However, sweet foods in the latter were generally healthy core foods (e.g., fruit, vegetables and dairy). Similar work concerning dietary taste patterns and diet quality needs exploring in adolescents. This could help in understanding adolescents' dietary choices and behaviors in relation to their taste preferences, which could aid in designing interventions or educational programs tailoring adolescents' food choices by their taste preferences. Also, findings could help food producers (e.g., school canteens, caregivers, food industries) in promoting more varieties of foods and tastes.

## Conclusion

Our findings have characterized diets of UK adolescents by taste, a key factor influencing food choice. We found that energy intake was dominated by sweet tasting and neutral foods. Protein and vegetable intakes were linked to an increased intake of savory-tasting foods. Individuals in this cohort had limited intakes of foods with a sour taste. Adolescents' dietary intakes may be driven by their taste preferences which may, in turn, be important determinants of later health as they grow into adulthood.

## Data Availability Statement

The data analyzed in this study is subject to the following licenses/restrictions: not made available to the public. Requests to access these datasets should be directed to https://ukdataservice.ac.uk/.

## Ethics Statement

Ethics approval for the online survey was obtained from the University of Leeds MEEC 19-039. The patients/participants provided their written informed consent to participate in this study.

## Author Contributions

AB identified the aim and methods of the study, carried out statistical and data analysis, wrote the first draft of the manuscript, and revised all subsequent drafts. MZ, CE, and JC revised and assessed the designed research question, statistical analysis, and all versions of the manuscript. All authors read and approved the submitted version.

## Funding

AB is a Ph.D. student in receipt of a scholarship from King Abdulaziz University, Jeddah, Saudi Arabia.

## Conflict of Interest

JC is Director of Dietary Assessment Ltd. The remaining authors declare that the research was conducted in the absence of any commercial or financial relationships that could be construed as a potential conflict of interest.

## Publisher's Note

All claims expressed in this article are solely those of the authors and do not necessarily represent those of their affiliated organizations, or those of the publisher, the editors and the reviewers. Any product that may be evaluated in this article, or claim that may be made by its manufacturer, is not guaranteed or endorsed by the publisher.
