# Corrigendum: Characterizing adolescents' dietary intake by taste: Results from the UK National Diet and Nutrition Survey

**DOI:** 10.3389/fnut.2022.1046893

**Published:** 2022-11-16

**Authors:** Areej Bawajeeh, Michael A. Zulyniak, Charlotte E. L. Evans, Janet E. Cade

**Affiliations:** ^1^Nutritional Epidemiology Group, School of Food Science and Nutrition, University of Leeds, Leeds, United Kingdom; ^2^Department of Food and Nutrition, Faculty of Human Sciences and Design, King Abdulaziz University, Jeddah, Saudi Arabia

**Keywords:** taste, dietary taste, NDNS, adolescents, taste perception

In the published article, there was an error in Table 2 as published. The rows for “neutral” and “savory” tastes are not separated in the published article, and are instead all combined under “neutral taste.” Moreover, the row headings for the rest of the table are all stated as “neutral taste” rather than being specified for the other tastes (savory, salty, and bitter). The corrected Table 2 and its caption appear below.

**Table 2:** Characteristics of adolescents' dietary intakes by the quintiles (Q) weight of foods consumed as a percentage of the total food weight.

	**Quintiles of sweet-tasting foods as percentage of the total food weight** (%)							
	**Q1 (*n* = 57)**	**Q2 (*n* = 57)**	**Q3 (*n* = 57)**	**Q4 (*n* = 57)**	**Q5 (*n* = 56)**	**%Diff Q1&Q5**	Coeff. (95% CI)^*^	***P*** **-trend**
	**7–31%**	**31–37%**	**37–43%**	**43–50%**	**50-73%**			
Energy (kcal/d)	1,449 (1,330, 1,569)	1,574 (1,428, 1,721)	1,696 (1,564, 1,828)	1,750 (1,620, 1,879)	1,738 (1,619, 1,858)	20%	10 (6, 15)	<0.01
Carbohydrate (g/d)	183 (169, 198)	208 (186, 231)	223 (209, 237)	234 (216, 253)	250 (235, 266)	37%	2 (1.5, 3)	<0.01
Protein (g/d)	62 (56, 67)	62 (56, 68)	65 (59, 72)	69 (6276)	58 (53, 64)	−6%	0.02 (−0.2, 0.2)	0.83
Fat (g/d)	57 (51, 63)	60 (54, 66)	66 (59, 74)	65 (59, 71)	62 (56, 68)	9%	0.3 (0.03, 0.5)	0.02
Total sugars (g/d)	54 (48, 61)	71 (62, 80)	89 (80, 99)	96 (87, 105)	116 (105, 127)	115%	2 (1.5, 2)	<0.01
Free sugars (g/d)	34 (28, 40)	47 (40, 54)	61 (51, 72)	63 (51, 74)	84 (72, 97)	147%	1.5 (1, 2)	<0.01
Fibre (g/d)	14 (13, 15)	16 (14, 18)	15 (13, 16)	16 (14, 17)	15 (14, 17)	7%	0.04 (−0.01, 0.1)	0.14
Saturated fat (g/d)	19 (17, 21)	22 (19, 25)	24 (21, 28)	26 (23, 28)	25 (22, 28)	32%	0.2 (0.1, 0.3)	<0.01
Sodium (mg/d)	1,791 (1,580, 2,003)	1,772 (1,584, 1,961)	1,983 (1,771, 2,195)	1,942 (1,769, 2,114)	1,651 (1,456, 1,846)	−8%	1 (−7, 8)	0.86
Fruit (g/d)	55 (37, 73)	52 (33, 72)	69 (50, 88)	71 (42, 100)	88 (58, 118)	60%	1.2 (0.3, 2)	<0.01
Fruit juice (g/d)	57 (25, 89)	72 (32, 112)	88 (60, 117)	82 (44, 120)	149 (73, 225)	161%	2 (0.3, 4)	0.02
Brassica vegetables (g/d)	12 (6, 19)	12 (7, 17)	16 (7, 24)	10 (4, 16)	10 (5, 15)	−17%	−0.04 (−0.2, 0.2)	0.66
Other vegetables (g/d)	87 (69, 105)	97 (77, 117)	73 (62, 84)	106 (83, 130)	73 (57, 90)	−16%	−0.3 (−1, 0.4)	0.42
Meat & poultry (g/d)	72 (56, 89)	55 (44, 65)	72 (51, 93)	59 (47, 72)	45 (35, 55)	−38%	−0.5 (−1, −0.3)	0.03
Processed meats (g/d)	25 (17, 33)	26 (17, 35)	29 (21, 36)	28 (19, 36)	18 (11, 26)	−28%	−0.1 (−0.4, 0.3)	0.66
Cheese (g/d)	18 (12, 24)	22 (15, 28)	16 (11, 22)	17(12, 23)	18 (13, 23)	0%	−0.1 (−0.3, 0.1)	0.55
	**Quintiles of neutral-tasting foods as percentage of the total food weight (%)**							
	**Q1 (*****n** =* **57)**	**Q2 (*****n** =* **57)**	**Q3 (*****n** =* **57)**	**Q4 (*****n** =* **57)**	**Q5 (*****n** =* **56)**	**%Diff Q1&Q5**	**Coeff. (95% CI)** ^*^	***P-*** **trend**
	**9–26%**	**26–33%**	**33–38%**	**38–46%**	**46–78%**			
Energy (kcal/d)	1,772 (1,647, 1,898)	1,721 (1,580, 1,863)	1,644 (1,537, 1,751)	1,601 (1,445, 1,757)	1,436 (1,317, 1,555)	−19%	−10 (−15, −5)	<0.01
Carbohydrate (g/d)	243 (225, 261)	228 (210, 247)	218 (204, 233)	211 (190, 233)	191 (173, 209)	−21%	−2 (−2, −1)	<0.01
Protein (g/d)	60 (54, 65)	69 (62, 76)	66 (60, 72)	63 (56, 70)	58 (53, 63)	−3%	−0.1 (−0.4, 0.1)	0.25
Fat (g/d)	69 (61, 76)	64 (56, 71)	61 (57, 66)	62 (55, 69)	55 (49, 60)	−20%	−0.4 (−1, −0.1)	0.02
Total sugars (g/d)	111 (98, 124)	93 (83, 103)	80 (72, 87)	77 (66, 87)	59 (52, 67)	−47%	−1 (−2, −1)	<0.01
Free sugars (g/d)	82 (69, 95)	63 (52, 74)	52 (44, 59)	51 (41, 60)	38 (31, 44)	−54%	−1 (−2, −1)	<0.01
Fibre (g/d)	15 (13, 16)	16 (14, 17)	16 (14, 17)	15 (13, 17)	14 (13, 16)	−7%	−0.02 (−0.1,0.03)	0.48
Saturated fat (g/d)	28 (24, 32)	25 (21, 28)	22 (21, 24)	22 (19, 25)	18 (16, 20)	−36%	−0.3 (−0.4, −0.2)	<0.01
Sodium (mg/d)	1,845 (1,605, 2,085)	2,013 (1,848, 2178)	1,841 (1,611, 2,072)	1,801 (1,604, 1,997)	1,648 (1,473, 1,823)	−11%	−7 (−15, 1)	0.07
Fruit (g/d)	64 (39, 89)	72 (50, 93)	68 (40, 95)	64 (45, 84)	60 (42, 79)	−6%	−0.1 (−1, 1)	0.73
Fruit juice (g/d)	131 (56, 206)	90 (44, 137)	86 (53, 118)	67 (34, 100)	66 (32, 101)	−50%	−1 (−4, 1)	0.19
Brassica vegetables (g/d)	10 (5, 15)	13 (7, 20)	12 (5, 20)	12 (6, 17)	12 (6, 19)	20%	0.01 (−0.2, 0.2)	0.94
Other vegetables (g/d)	77 (60, 94)	106 (80, 132)	84 (73, 95)	82 (64, 100)	89 (69, 108)	16%	−0.1 (−1, 1)	0.81
Meat and poultry (g/d)	44 (34, 53)	63 (51, 75)	66 (45, 86)	70 (53, 86)	62 (49, 76)	41%	0.4 (−0.1, 1)	0.13
Processed meats (g/d)	32 (22, 43)	32 (24, 40)	20 (14, 26)	25 (17, 32)	18 (11, 25)	−44%	−0.4 (−1, −0.1)	<0.01
Cheese (g/d)	23 (16, 30)	18 (13, 23)	20 (13, 26)	17 (12, 22)	16 (10, 21)	−30%	−0.2 (−0.4, 0.03)	0.10
	**Quintiles of savory-tasting foods as percentage of the total food weight (%)**							
	**Q1 (*****n** =* **57)**	**Q2 (*****n** =* **57)**	**Q3 (*****n** =* **57)**	**Q4 (*****n** =* **57)**	**Q5 (*****n** =* **56)**	**%Diff Q1&Q5**	**Coeff. (95% CI)** ^*^	***P*** **-trend**
	**0–7%**	**7–10%**	**10–12%**	**12–16%**	**16–27%**			
Energy (kcal/d)	1,678 (1,565, 1,791)	1,698 (1,566, 1,831)	1,609 (1,476, 1,741)	1584 (1,443, 1,725)	1,581 (1,433, 1,730)	−6%	−9 (−19, 0.4)	0.06
Carbohydrate (g/d)	233 (216, 249)	231 (213, 249)	220 (201, 240)	207 (188, 227)	200 (181, 220)	−14%	−3 (−4, −1)	<0.01
Protein (g/d)	58 (53, 62)	63 (58, 69)	61 (56, 66)	65 (58, 72)	66 (59, 73)	14%	0.4 (−0.01, 1)	0.05
Fat (g/d)	63 (57, 69)	64 (57, 70)	60 (53, 66)	60 (54, 66)	63 (55, 70)	0%	−0.2 (−1, 1)	0.92
Total sugars (g/d)	100 (88, 113)	89 (78, 100)	89 (76, 102)	71 (62, 80)	70 (61, 79)	−30%	−2 (−3, −1)	<0.01
Free sugars (g/d)	69 (57, 82)	59 (50, 69)	65 (52, 77)	45 (37, 53)	47 (37, 57)	−32%	−2 (−2, −1)	<0.01
Fibre (g/d)	16 (14, 17)	16 (15, 18)	13 (12, 15)	15 (14, 17)	14 (12, 16)	−13%	−0.1 (−0.2, 0.01)	0.07
Saturated fat (g/d)	25 (21, 28)	24 (21, 26)	23 (20, 25)	21 (19, 24)	22 (19, 26)	−12%	−0.1 (−0.4, 0.2)	0.42
Sodium (mg/d)	1,762 (1,637, 1,888)	1,810 (1,639, 1,981)	1,734 (1,553, 1,915)	1,925 (1,690, 2,160)	1,840 (1,600, 2,080)	4%	7 (−9, 23)	0.37
Fruit (g/d)	100 (70, 129)	85 (61, 109)	50 (33, 66)	48 (35, 60)	53 (30, 75)	−47%	−3 (−5, −1)	<0.01
Fruit juice (g/d)	97 (53, 140)	133 (65, 202)	77 (50, 104)	53 (33, 73)	84 (31, 136)	−13%	−3 (−7, 1)	0.12
Brassica vegetables (g/d)	7 (3, 11)	17 (8, 25)	8 (3, 12)	13 (6, 21)	13 (8, 19)	86%	0.2 (−0.2, 1)	0.39
Other vegetables (g/d)	65 (51, 80)	91 (69, 112)	82 (63, 101)	97 (81, 113)	97 (76, 118)	49%	2 (0.2, 3)	0.03
Meat & poultry (g/d)	38 (29, 46)	59 (48, 71)	60 (48, 71)	71 (54, 88)	72 (54, 90)	90%	2 (0.4, 3)	0.01
Processed meats (g/d)	21 (14, 27)	26 (17, 35)	20 (14, 26)	22 (15, 29)	35 (25, 44)	67%	1 (0.2, 2)	0.01
Cheese (g/d)	18 (13, 23)	21 (15, 27)	19 (15, 24)	15 (9, 20)	20 (13, 27)	11%	0.1 (−1, 1)	0.85
	**Quintiles of salty-tasting foods as percentage of the total food weight (%)**							
	**Q1 (*****n** =* **57)**	**Q2 (*****n** =* **57)**	**Q3 (*****n** =* **57)**	**Q4 (*****n** =* **57)**	**Q5 (*****n** =* **56)**	**%Diff Q1&Q5**	**Coeff. (95%CI)** ^*^	***P*** **-trend**
	**0–3%**	**3–6%**	**6–8%**	**8–11%**	**11–31%**			
Energy (kcal/d)	1,617 (1,474, 1,760)	1,655 (1,493, 1,816)	1,579 (1,439, 1,720)	1,649 (1,536, 1,762)	1,621 (1,499, 1,742)	0%	2 (−9, 12)	0.72
Carbohydrate (g/d)	214 (195, 234)	226 (201, 250)	210 (194, 227)	226 (209, 244)	206 (191, 220)	−4%	−0.4 (−2, 1)	0.50
Protein (g/d)	67 (59, 74)	66 (60, 73)	61 (55, 67)	59 (54, 63)	63 (57, 68)	−6%	−0.3 (−1,.2)	0.26
Fat (g/d)	60 (54, 67)	61 (54, 67)	60 (52, 68)	62 (57, 68)	66 (59, 73)	10%	1 (−0.1, 1)	0.07
Total sugars (g/d)	83 (72, 94)	84 (72, 96)	87 (74, 100)	89 (77, 101)	69 (61, 77)	−17%	−0.7 (−2, 0.1)	0.08
Free sugars (g/d)	53 (42, 64)	53 (42, 63)	63 (51, 75)	64 (52, 75)	47 (40, 54)	−11%	−0.2 (−1, 1)	0.65
Fibre (g/d)	15 (14, 17)	15 (13, 17)	14 (13, 16)	15 (13, 16)	15 (14, 17)	0%	−0.02 (−0.1, 0.1)	0.80
Saturated fat (g/d)	22 (19, 25)	22 (19, 25)	22 (19, 26)	23 (21, 25)	25 (22, 29)	14%	0.3 (0.02, 1)	0.03
Sodium (mg/d)	1,770 (1,545, 1,996)	1,717 (1,511, 1,923)	1,711 (1,523, 1,898)	1,825 (1,642, 2,008)	2,101 (1,893, 2,309)	19%	22 (4.5, 40)	0.01
Fruit (g/d)	66 (49, 83)	92 (62, 122)	64 (43, 85)	66 (44, 89)	35 (23, 47)	−47%	−2 (−4, −1)	<0.01
Fruit Juice (g/d)	70 (35, 104)	66 (35, 98)	111 (50, 172)	126 (67, 184)	60 (31, 89)	−14%	−0.5 (−3, 2)	0.73
Brassica vegetables (g/d)	19 (10, 28)	11 (6, 15)	12 (6, 19)	10 (6, 15)	7 (3, 11)	−63%	−1 (−1, −0.2)	<0.01
Other vegetables (g/d)	101 (81, 120)	91 (71, 111)	91 (74, 107)	78 (56, 100)	77 (62, 92)	−24%	−2 (−3, −0.3)	0.01
Meat & poultry (g/d)	75 (53, 96)	72 (57, 87)	56 (46, 66)	57 (47, 67)	42 (33, 52)	−44%	−2 (−3, −1)	<0.01
Processed meats (g/d)	14 (8, 21)	22 (14, 31)	22 (15, 28)	28 (22, 35)	40 (30, 49)	186%	2 (1, 2)	<0.01
Cheese (g/d)	10 (6, 14)	15 (10, 19)	18 (14, 22)	19 (14, 24)	33 (26, 41)	230%	1 (1, 2)	<0.01
	**Quintiles of bitter-tasting foods as percentage of the total food weight (%)**							
	**Q1 (*****n** =* **88)**	**Q2 (*****n** =* **26)**	**Q3 (*****n** =* **57)**	**Q4 (*****n** =* **57)**	**Q5 (*****n** =* **56)**	**%Diff Q1&Q5**	**Coeff. (95%CI)** ^*^	***P-*** **trend**
	**0%**	<**1–1%**	**1–4%**	**4–7%**	**7–27%**			
Energy (kcal/d)	1,570 (1,454, 1,686)	1,808 (1,584, 2,032)	1,607 (1,501, 1,713)	1,673 (1,517, 1,828)	1,585 (1,475, 1,696)	1%	−3 (−15, 9)	0.62
Carbohydrate (g/d)	213 (196, 231)	245 (211, 278)	219 (207, 231)	215 (195, 235)	210 (192, 228)	−1%	−1 (−3, 1)	0.30
Protein (g/d)	58 (54, 62)	73 (62, 84)	60 (53, 67)	67 (61, 74)	63 (57, 69)	9%	0.2 (−0.4, 1)	0.41
Fat (g/d)	60 (55, 65)	67 (59, 75)	61 (55, 66)	66 (58, 74)	58 (54, 63)	−3%	−0.3 (−1, 0.2)	0.28
Total sugars (g/d)	81 (70, 93)	91 (75, 107)	86 (76, 97)	78 (67, 89)	82 (72, 93)	1%	−0.2 (−1, 1)	0.77
Free sugars (g/d)	57 (47, 67)	57 (44, 71)	58 (47, 69)	54 (44, 64)	54 (43, 65)	−5%	−0.3 (−1, 1)	0.59
Fibre (g/d)	14 (13, 15)	18 (15, 21)	15 (14, 16)	15 (14, 17)	15 (13, 16)	7%	−0.02 (−0.2, 0.2)	0.86
Saturated fat (g/d)	23 (20, 25)	26 (22, 30)	21 (20, 23)	25 (21, 28)	21 (19, 23)	−9%	−0.2 (−0.4, 0.1)	0.15
Sodium (mg/d)	1,722 (1,590, 1,855)	2,036 (1,763, 2,309)	1,779 (1,535, 2,023)	1,906 (1,660, 2,152)	1,803 (1,664, 1,941)	5%	1 (−15, 18)	0.86
Fruit (g/d)	69 (50, 89)	85 (38, 132)	72 (49, 96)	54 (36, 72)	59 (39, 79)	−14%	−1 (−4, 1)	0.34
Fruit juice (g/d)	86 (56, 115)	118 (60, 176)	120 (50, 190)	69 (41, 97)	61 (21, 102)	−29%	−4 (−8, 1)	0.11
Brassica vegetables (g/d)	6 (2, 10)	8 (2, 13)	17 (10, 25)	12 (6, 18)	15 (9, 21)	150%	1 (−0.1, 1)	0.07
Other vegetables (g/d)	63 (51, 74)	112 (73, 151)	97 (78, 115)	84 (68, 100)	104 (85, 124)	65%	3 (1, 5)	0.01
Meat & poultry (g/d)	53 (42, 65)	55 (37, 73)	64 (45, 84)	65 (53, 76)	66 (51, 81)	25%	1 (−1, 2)	0.41
Processed meats (g/d)	21 (16, 26)	43 (28, 58)	20 (14, 26)	30 (21, 39)	22 (16, 29)	5%	−0.1 (−1, 1)	0.81
Cheese (g/d)	17 (13, 22)	23 (15, 32)	15 (9, 20)	25 (18, 31)	15 (11, 19)	−12%	−0.2 (−1, 0.3)	0.51

* Change in nutrient/food per % increase in taste.

Q1–Q5 = quintiles 1 (lowest quintile)- quintiles 5 (highest quintile). Each quintile represents: (1) number of adolescents (n); although they are in the same size it is different individuals; (2) proportion of food tastes (%).

A corrections has been made in **Results, Neutral-Tasting Foods**. The paragraph previously stated:

“Energy, carbohydrate, sugars, total fat and saturated fats all showed significant negative linear trends with increasing neutral-tasting foods. Energy intake decreased by 19% from the lowest to the highest quintile and there was a statistically significant negative trend of lower energy intake by 10 kcal/d (95% CI −15, −5; *P* < 0.001) for each increase in the proportion of neutral-tasting foods. Individuals in the highest quintile of neutral-tasting foods had lower carbohydrate (21%), total sugars (47%), and free sugars (54%) compared to those in the lowest quintile. Total fat and saturated fats intakes also showed negative overall trends of lower intakes with higher consumption of neutral-tasting foods. Processed meats consumption was 44% higher in the highest compared to the lowest quintile of neutral-tasting foods; with an overall significant trend (*P* < 0.01) per each percentage increase in neutral-tasting foods.”

The corrected paragraph appears below:

“Energy, carbohydrate, sugars, total fat and saturated fats all showed significant negative linear trends with increasing neutral-tasting foods. Energy intake decreased by 19% from the lowest to the highest quintile and there was a statistically significant negative trend of lower energy intake by 10 kcal/d (95% CI −15, −5; *P* < 0.001) for each increase in the proportion of neutral-tasting foods. Individuals in the highest quintile of neutral-tasting foods had lower carbohydrate (21%), total sugars (47%), and free sugars (54%) compared to those in the lowest quintile. Total fat and saturated fats intakes also showed negative overall trends of lower intakes with higher consumption of neutral-tasting foods. Processed meats consumption was 44% lower in the highest compared to the lowest quintile of neutral-tasting foods; with an overall significant trend (*P* < 0.01) per each percentage increase in neutral-tasting foods.”

The authors apologize for these errors and state that they do not change the scientific conclusions of the article in any way. The original article has been updated.

## Publisher's note

All claims expressed in this article are solely those of the authors and do not necessarily represent those of their affiliated organizations, or those of the publisher, the editors and the reviewers. Any product that may be evaluated in this article, or claim that may be made by its manufacturer, is not guaranteed or endorsed by the publisher.

