# Supplementary material for: Characterizing Adolescents' Dietary Intake by Taste: Results From the UK National Diet and Nutrition Survey
Source: Front Nutr. 2022 Jun 20;9:893643. doi: 10.3389/fnut.2022.893643 (PMC9251474; doi:10.3389/fnut.2022.893643)
Supplement: Supplementary file 1 [file Data_Sheet_1.docx]

Table S1. Examples of the grouped food list under the main and subsidiary food groups

| Main food groups | Sub food groups | Examples |
| --- | --- | --- |
| Cereals and cereal products | Breakfast cereals, bread, pasta, rice, and pizza | Breakfast cereals, Plain pasta/rice, White bread |
|  | Buns cakes, pastries, and puddings | Plain scones, Eclairs, doughnuts, Lemon meringue pie |
|  | Biscuits | Chocolate biscuits, Digestives, cheese flavoured biscuits |
| Vegetables, beans, and seeds | Root vegetables | Carrots, potato, radish |
|  | Leafy type vegetables | Mixed leaf salad, lettuce, rocket, cabbage, spinach |
|  | Other vegetables | Cucumber, aubergine, mushrooms |
|  | Vegetable based dishes, and salads | Vegetable samosa, pakora, cauliflower cheese, guacamole |
|  | Beans, lentils, and seeds | Canned baked beans, hummus, seeds (e.g., sunflower, pumpkin) |
| Fruit | Fruit | Banana, kiwi, grapefruit |
| Meats, poultry, eggs and seafoods | Meats/chicken | Beef, chicken (grilled, roasted), processed meat (e.g., sausages) |
|  | Meats/chicken meals, pastry, and eggs | Burgers, kebab, fried egg, boiled eggs/poached |
|  | Seafood | White fish, sushi, tinned fish (e.g., tuna canned in oil) |
| Dairy products and fats | Milk, milk products and alternatives | Whole milk, plain yogurt, alternatives milk (e.g., almond, soy) |
|  | Cheese | Feta cheese, cheddar cheese, cheese spreads |
| Beverages | Juices and fizzy drinks | Ready to drink fruit juice (carton/can), Lemon juice sweetened, |
|  | Wine, beer, and alcoholic drinks | White wine, Beer, lager, Liqueurs |
|  | Coffee and tea | Coffee drinks with milk (e.g., latte, cappuccino), Black coffee, tea |
|  | Dry weight beverages | Drinks made from powder (e.g., sweetened drinking chocolate) |
| Miscellaneous | Sugar, sugar confectionery and chocolate confectionery | Boiled sweets and gums, Milk chocolate, Dark chocolate (70% cocoa) |
|  | Preserves, and spreads | Honey, jam, chocolate spread, Peanut butter smooth/crunchy with added sugar, Peanut butter no added sugar |
|  | Savoury snacks (e.g., crisps and nuts) | Crisps, Unsalted nuts, Honey roasted peanuts |
|  | Soups | Tomato soup, Chicken soup, Vegetable soup |
|  | Salt, herbs, spices, pickles, pastes, and sauces | Chinese sweet & sour sauce, White sauce, Gravy |

Table S2. Explanation for each taste as provided in the online foods taste classification survey

| Taste | Definition explained |
| --- | --- |
| **Sweet taste** | **the sense of sweetness of sugar on your tongue** |
| **Salty taste** | **the sense of saltiness from salt on your tongue** |
| **Sour taste** | **the sense of sourness of lemon on your tongue** |
| **Bitter taste** | **the sense of a sharp, potentially disagreeable taste like the bitterness of caffeine** |
| **Savoury** | **often described as ‘broth-like’ or ‘meaty’. It is like the taste found in Japanese food and soy sauce** |
| **Neutral taste** | **the sense of little or no specific taste** |

Table S3. Removed foods after the pilot

| Sweet-tasting foods | |
| --- | --- |
| Cereals and cereal products | **Fruit** |
| Breakfast cereal with chocolate or honey or fruit  High fibre breakfast cereal with chocolate or honey or fruit  Sweet pastries and breads e.g., currant bun, danish pastry, scotch pancake  Eclairs, doughnuts, cream, or iced bun  Puddings e.g., sponge, trifle, bread, and butter pudding  Cakes and sweet muffins  Custard, mousse, dairy desserts, rice pudding, jelly  Custard, mousse, dairy desserts, rice pudding, jelly  Chocolate biscuits, chocolate chip cookies  Cream, jam filled or iced biscuits  Biscuits with fruits or currants  Cereal bar with fruit  Cereal bars (e.g., Kellogg's rice Krispy, coco pops, Frosties), cereal bars with oats | Apples, pears  Peaches, nectarines, plums, apricots  Mango, papaya  Banana  Melon  Strawberries, raspberries  Cherries  Grapes  Pineapple  Dried mixed fruit  Mixed fruit puree, compote, canned fruit  Fruit bars, sticks  Desiccated coconut sweetened  Condensed milk, coffee creamer liquid  Condensed milk, coffee creamer liquid  Fruit yogurt and fromage frais |
|  | **Vegetables** |
|  | Carrots  Sweet potatoes  Sweetcorn  Peppers (red, green, yellow |
| *Dairy* products | **Beverages** |
| Ice cream  Condensed milk, coffee creamer liquid  Milkshake, hot chocolate, flavoured milk  Fruit yogurt and fromage frais | Mixed fruit smoothie, sweetened  Ready to drink fruit juice (carton/can)  Apple juice unsweetened  Concentrated soft drinks/squash (e.g., Ribena)  Fizzy drinks (e.g., lemonade)  Alcoholic fruit drinks (e.g., cocktails)  Drinks made with powder (e.g., drinking chocolate, mocha, milkshake) |
| *Mi*scellaneous |  |
| Sugar, syrups, sweeteners  Boiled sweets and gums  Milk chocolate  Honey, jam, chocolate spread |  |
| Salty-tasting foods | |
| Cereals and cereal products | **Miscellaneous** |
| Salted biscuits e.g., Tuc, Ritz | Crisps and savoury snacks (e.g., tortilla chips), Bombay mix / chevda  Salted popcorn, salted nuts  Salt |
| Bitter-tasting foods | |
| Beverages | |
| Beer, lager  Black coffee & Black tea | |
| Savoury/Umami | |
| Meats, poultry, eggs and seafoods | |
| Meat or chicken-based dishes e.g., Lasagne, cottage pie, hotpot | |
| Neutral-tasting foods | |
| Cereals and cereal products: Plain pasta, noodles, rice | |

**Table S4.** Validity checking of the taste classification

| Food | Our work (Values= respondents) | | | | | | Others’ work (Values= mean intensity) | | | | | Validity outcome |
| --- | --- | --- | --- | --- | --- | --- | --- | --- | --- | --- | --- | --- |
|  | Sweet | Salty | Sour | Bitter | Savoury | Neutral | Sweet | Salty | Sour | Bitter | Savoury |  |
| Fruit yogurt and fromage frais | 15 | 0 | 3 | 0 | 1 | 0 | 34 | 3 | 27 | 1 | 1 | Agreed |
| Breakfast cereal (e.g. cornflakes, multigrain) without honey, chocolate, fruit | 50 | 4 | 1 | 1 | 6 | 38 | 14 | 9 | 2 | 1 | 2 |  |
| Pasta with tomato or cheese sauces | 16 | 31 | 2 | 1 | 42 | 8 | 9 | 29 | 11 | 0 | 25 |  |
| Pot noodle | 2 | 65 | 0 | 0 | 31 | 1 | 9 | 45 | 3 | 1 | 37 |  |
| Rice or risotto dishes (e.g. fried rice with chicken or egg) | 2 | 35 | 0 | 0 | 55 | 9 | 6 | 39 | 2 | 1 | 27 |  |
| Pizza | 12 | 48 | 1 | 1 | 32 | 5 | 12 | 32 | 11 | 1 | 29 |  |
| Plain scones | 52 | 7 | 1 | 1 | 8 | 31 | 44 | 12 | 1 | 1 | 1 |  |
| Fruit pies and crumbles | 98 | 0 | 2 | 0 | 0 | 0 | 54 | 11 | 14 | 1 | 2 |  |
| Beef | 2 | 10 | 0 | 0 | 84 | 4 | 4 | 27 | 3 | 2 | 35 |  |
| Lamb | 6 | 8 | 1 | 1 | 78 | 5 | 11 | 38 | 5 | 2 | 36 |  |
| Chicken, duck | 4 | 8 | 0 | 0 | 75 | 12 | 5 | 28 | 2 | 1 | 28 |  |
| salami | 3 | 68 | 0 | 1 | 26 | 1 | 3 | 51 | 19 | 1 | 21 |  |
| corned beef | 3 | 68 | 0 | 1 | 26 | 1 | 4 | 43 | 8 | 1 | 23 |  |
| Processed meat e.g. sausages, pastrami | 3 | 68 | 0 | 1 | 26 | 1 | 5 | 46 | 10 | 1 | 23 |  |
| Smoked bacon | 3 | 70 | 1 | 1 | 25 | 0 | 6 | 67 | 6 | 0 | 15 |  |
| Unsmoked bacon | 1 | 69 | 1 | 1 | 26 | 2 | 6 | 67 | 6 | 0 | 15 |  |
| Burgers, kebab | 2 | 34 | 0 | 0 | 62 | 2 | 10 | 31 | 11 | 1 | 27 |  |
| Chicken kebab | 8 | 41 | 1 | 0 | 42 | 9 | 4 | 37 | 6 | 1 | 17 |  |
| nuggets | 8 | 41 | 1 | 0 | 42 | 9 | 7 | 35 | 3 | 1 | 21 |  |
| Meat or chicken based spicy dishes e.g. curry, biryani | 4 | 25 | 4 | 5 | 61 | 1 | 6 | 37 | 2 | 2 | 32 |  |
| Cheese quiche, souffle, scotch egg, vegetable and cheese pie | 7 | 46 | 0 | 2 | 39 | 6 | 6 | 54 | 10 | 1 | 23 |  |
| Eggs boiled, poached | 6 | 9 | 0 | 0 | 33 | 52 | 5 | 10 | 2 | 1 | 8 |  |
| White fish steamed, grilled, baked, fried | 6 | 16 | 1 | 0 | 28 | 51 | 2 | 13 | 3 | 2 | 12 |  |
| Fish curry, prawn curry | 7 | 32 | 5 | 3 | 49 | 5 | 6 | 37 | 2 | 2 | 32 |  |
| Oily fish meals e.g. salmon, herring, mackerel, fresh tuna | 5 | 33 | 0 | 2 | 52 | 8 | 3 | 29 | 10 | 1 | 26 |  |
| Tinned fish e.g. tuna canned in oil | 5 | 37 | 1 | 5 | 39 | 13 | 3 | 37 | 19 | 2 | 31 |  |
| Tinned fish canned in water | 5 | 34 | 1 | 4 | 37 | 20 | 4 | 25 | 16 | 2 | 22 |  |
| Mackerel in tomato sauce | 17 | 32 | 5 | 5 | 34 | 7 | 3 | 42 | 8 | 1 | 44 |  |
| Whole milk | 57 | 1 | 4 | 1 | 3 | 34 | 12 | 3 | 4 | 1 | 1 |  |
| Semi-skimmed milk | 48 | 0 | 3 | 1 | 3 | 45 | 12 | 3 | 4 | 1 | 1 |  |
| Skimmed milk | 31 | 0 | 8 | 0 | 2 | 60 | 14 | 2 | 5 | 1 | 1 |  |
| Plain yogurt, sour cream, buttermilk | 11 | 1 | 67 | 4 | 6 | 12 | 4 | 4 | 54 | 2 | 2 |  |
| Mozzarella | 26 | 26 | 6 | 1 | 16 | 26 | 2 | 9 | 7 | 1 | 3 |  |
| Radish | 6 | 4 | 14 | 54 | 8 | 14 | 5 | 1 | 6 | 7 | 3 |  |
| Celeriac | 27 | 7 | 11 | 13 | 18 | 24 | 12 | 3 | 3 | 3 | 3 |  |
| Mixed leaf salad, lettuce, rocket | 9 | 1 | 4 | 50 | 14 | 23 | 7 | 1 | 2 | 6 | 1 |  |
| Spring onions, chives, leeks | 16 | 5 | 14 | 29 | 26 | 10 | 5 | 2 | 3 | 11 | 2 |  |
| Spinach | 7 | 3 | 7 | 44 | 12 | 27 | 7 | 4 | 4 | 8 | 3 |  |
| Cabbage, Chinese leaves | 16 | 3 | 4 | 33 | 13 | 31 | 3 | 1 | 3 | 6 | 1 |  |
| Brussels sprouts | 7 | 2 | 7 | 58 | 16 | 10 | 8 | 5 | 2 | 19 | 9 |  |
| Courgette | 31 | 2 | 2 | 16 | 10 | 39 | 7 | 2 | 1 | 2 | 4 |  |
| Carrots | 15 | 0 | 0 | 0 | 2 | 2 | 15 | 2 | 2 | 1 | 7 |  |
| Crispy seaweed, seaweed wakame dried | 8 | 42 | 2 | 13 | 29 | 6 | 6 | 22 | 3 | 1 | 40 |  |
| Mushrooms | 9 | 5 | 0 | 4 | 57 | 25 | 7 | 5 | 3 | 2 | 18 |  |
| Peas | 74 | 1 | 0 | 4 | 6 | 15 | 15 | 4 | 2 | 1 | 5 |  |
| Potato chips/waffles or roast | 16 | 47 | 0 | 0 | 17 | 20 | 9 | 15 | 3 | 0 | 7 |  |
| Vegetable soup | 15 | 42 | 1 | 2 | 29 | 11 | 19 | 32 | 16 | 1 | 27 |  |
| Clear soup (e.g. consommé, stock cubes) | 3 | 63 | 0 | 2 | 25 | 7 | 10 | 39 | 6 | 3 | 21 |  |
| Plain biscuits e.g. digestives, shortbread | 83 | 9 | 0 | 0 | 4 | 5 | 51 | 11 | 0 | 3 | 2 |  |
| Cheese straws, cheese flavoured biscuits | 7 | 69 | 1 | 0 | 18 | 5 | 6 | 41 | 2 | 0 | 8 |  |
| Prawn crackers, papadums | 17 | 48 | 0 | 0 | 27 | 9 | 12 | 29 | 1 | 0 | 26 |  |
| Olives | 2 | 44 | 8 | 30 | 13 | 2 | 4 | 31 | 6 | 4 | 18 |  |
| Red wine | 13 | 1 | 29 | 34 | 15 | 8 | 8 | 1 | 46 | 38 | 2 |  |
| White wine | 38 | 1 | 29 | 20 | 4 | 8 | 12 | 1 | 45 | 21 | 1 |  |
| 70% proof spirits e.g. whisky | 13 | 1 | 13 | 44 | 13 | 14 | 22 | 2 | 12 | 25 | 1 |  |
| Coffee drinks with milk, unsweetened (e.g. latte, cappuccino) | 30 | 1 | 4 | 48 | 8 | 10 | 8 | 2 | 9 | 44 | 1 |  |
| Twiglets, pretzels | 0 | 62 | 1 | 6 | 28 | 3 | 6 | 45 | 1 | 0 | 4 |  |
| Unsalted nuts | 9 | 6 | 3 | 3 | 50 | 30 | 7 | 8 | 1 | 6 | 9 |  |
| Honey roasted peanuts | 88 | 5 | 0 | 2 | 5 | 0 | 42 | 11 | 1 | 4 | 2 |  |
| Curry sauce/ curry paste | 18 | 21 | 4 | 7 | 48 | 3 | 31 | 33 | 22 | 1 | 22 |  |
| Pesto | 14 | 31 | 4 | 9 | 36 | 7 | 5 | 57 | 12 | 2 | 23 |  |
| Marmite | 0 | 46 | 2 | 17 | 32 | 2 | 11 | 62 | 23 | 23 | 46 |  |
| Mustard | 2 | 7 | 17 | 47 | 23 | 4 | 6 | 37 | 47 | 10 | 5 |  |
| French salad dressing | 22 | 16 | 41 | 6 | 9 | 6 | 10 | 41 | 56 | 2 | 11 |  |
| Eclairs, doughnuts, cream or iced bun | 19 | 0 | 0 | 0 | 0 | 0 | 44 | 10 | 1 | 1 | 2 |  |
| Crisps and savoury snacks (e.g. tortilla chips), Bombay mix / chevda | 1 | 15 | 1 | 0 | 2 | 0 | 9 | 45 | 5 | 2 | 13 |  |
| Apples, pears | 17 | 0 | 1 | 1 | 0 | 0 | 27 | 1 | 17 | 1 | 1 |  |
| Banana | 19 | 0 | 0 | 0 | 0 | 0 | 29 | 1 | 2 | 1 | 1 |  |
| Grapes | 18 | 0 | 0 | 1 | 0 | 0 | 30 | 1 | 25 | 2 | 0 |  |
| Meat or chicken-based dishes e.g. Lasagne, cottage pie, hotpot | 1 | 2 | 0 | 0 | 15 | 1 | 12 | 42 | 8 | 1 | 32 |  |
| Biscuits with fruits or currants | 17 | 0 | 1 | 0 | 1 | 0 | 30 | 7 | 5 | 1 | 1 |  |
| Milkshake, hot chocolate, flavoured milk | 18 | 0 | 0 | 0 | 0 | 0 | 37 | 6 | 2 | 7 | 0 |  |
| Custard, mousse, dairy desserts, rice pudding, jelly | 18 | 0 | 0 | 0 | 1 | 0 | 43 | 4 | 5 | 5 | 1 |  |
| Ice cream | 19 | 0 | 0 | 0 | 0 | 0 | 46 | 6 | 2 | 3 | 1 |  |
| Breakfast cereal with chocolate or honey or fruit | 19 | 0 | 0 | 0 | 0 | 0 | 41 | 12 | 1 | 5 | 0 |  |
| High fibre breakfast cereal with chocolate or honey or fruit | 17 | 0 | 0 | 0 | 0 | 1 | 23 | 9 | 3 | 1 | 1 |  |
| Black coffee | 0 | 0 | 0 | 17 | 2 | 0 | 2 | 1 | 9 | 63 | 1 |  |
| Black tea | 0 | 0 | 0 | 17 | 2 | 0 | 4 | 1 | 5 | 20 | 1 |  |
| Ready to drink fruit juice (carton/can) | 19 | 0 | 0 | 0 | 0 | 0 | 50 | 2 | 33 | 7 | 0 |  |
| Fizzy drinks (e.g. lemonade) | 17 | 0 | 2 | 0 | 0 | 0 | 41 | 1 | 23 | 5 | 0 |  |
| Beer, lager | 1 | 0 | 1 | 15 | 2 | 0 | 7 | 1 | 17 | 55 | 1 |  |
| Cakes and sweet muffins | 19 | 0 | 0 | 0 | 0 | 0 | 47 | 10 | 2 | 4 | 1 |  |
| Chocolate biscuits, chocolate chip cookies | 19 | 0 | 0 | 0 | 0 | 0 | 59 | 13 | 1 | 4 | 1 |  |
| Sweet peppers | 15 | 0 | 0 | 3 | 0 | 1 | 11 | 2 | 8 | 11 | 4 |  |
| Boiled sweets and gums | 17 | 0 | 0 | 0 | 0 | 0 | 46 | 2 | 19 | 2 | 0 |  |
| Jam | 19 | 0 | 0 | 0 | 0 | 0 | 74 | 3 | 19 | 1 | 0 |  |
| Honey | 19 | 0 | 0 | 0 | 0 | 0 | 76 | 4 | 1 | 3 | 0 |  |
| Marmite flavour rice cakes | 0 | 51 | 0 | 14 | 28 | 7 | 11 | 62 | 23 | 23 | 46 |  |
| Sushi | 9 | 20 | 2 | 2 | 57 | 10 | / | / | / | / | 28 |  |
| Tomato juice | 36 | 18 | 20 | 8 | 12 | 6 | 10 | 32 | 23 | 1 | 33 |  |
| Oyster, black bean, plum, satay sauce | 32 | 22 | 3 | 3 | 36 | 3 | 26 | 24 | 4 | 2 | 32 |  |
| Cheese spreads | 24 | 37 | 3 | 0 | 12 | 24 | 6 | 55 | 22 | 5 | 18 |  |
| Cottage cheese, ricotta, feta cheese | 8 | 51 | 20 | 2 | 7 | 12 | 9 | 37 | 22 | 2 | 24 |  |
| Vegetable samosa, pakora, pancake roll, bhaji | 13 | 34 | 2 | 2 | 46 | 4 | 16 | 24 | 1 | 1 | 12 |  |
| Fried plantain | 62 | 11 | 2 | 5 | 13 | 8 | 32 | 10 | 8 | 2 | 1 |  |
| Mango | 15 | 0 | 0 | 2 | 0 | 1 | 42 | 0 | 15 | 2 | 0 |  |
| Papaya | 15 | 0 | 0 | 2 | 0 | 1 | 31 | 1 | 2 | 2 | 2 |  |
| Sweetcorn | 19 | 0 | 0 | 0 | 0 | 0 | 15 | 2 | 0 | 0 | 0 |  |
| Coconut milk or cream | 82 | 0 | 4 | 0 | 2 | 12 | 25 | 4 | 9 | 1 | 1 |  |
| Coleslaw, Tzatziki, raita | 15 | 14 | 31 | 7 | 18 | 14 | 15 | 24 | 32 | 1 | 7 |  |
| Meats quiche, pastry and pies | 4 | 37 | 0 | 0 | 53 | 6 | 7 | 45 | 4 | 1 | 17 |  |
| Salted nuts | 2 | 17 | 0 | 0 | 0 | 0 | 12 | 32 | 1 | 4 | 11 |  |
| Egg fried, omelette, scrambled egg | 5 | 22 | 0 | 0 | 41 | 33 | 1 | 12 | 0 | 0 | 17 |  |
| Croissant plain | 72 | 6 | 0 | 0 | 7 | 14 | 13 | 19 | 2 | 1 | 1 | Disagreed |
| Tomatoes | 66 | 2 | 17 | 4 | 5 | 6 | 10 | 3 | 19 | 3 | 12 |  |
| Onions | 25 | 4 | 20 | 20 | 24 | 7 | 9 | 3 | 8 | 31 | 3 |  |
| Mashed potato | 28 | 17 | 1 | 0 | 13 | 41 | 7 | 44 | 3 | 1 | 15 |  |
| Baked potato | 26 | 11 | 2 | 0 | 17 | 45 | 15 | 44 | 8 | 1 | 18 |  |
| Canned baked beans | 74 | 11 | 0 | 1 | 10 | 4 | 18 | 28 | 6 | 1 | 16 |  |
| Canned tomatoes, pasta-tomato sauce, cook-in tomato sauce, tomato puree | 58 | 6 | 17 | 2 | 15 | 4 | 20 | 39 | 24 | 1 | 33 |  |
| Tomato ketchup | 73 | 8 | 8 | 0 | 10 | 0 | 28 | 29 | 42 | 1 | 22 |  |
| Mayonnaise, salad cream, Caesar salad dressing | 34 | 13 | 12 | 4 | 13 | 24 | 10 | 25 | 33 | 1 | 8 |  |
| White boiled rice | 0 | 2 | 0 | 0 | 0 | 17 | 3 | 2 | 2 | 2 | 4 | Neutral |
| Plain pasta | 0 | 2 | 0 | 0 | 0 | 17 | 3 | 3 | 2 | 1 | 1 |  |
| High fibre breakfast cereal (e.g. Weetabix, multigrain, bran) | 22 | 3 | 1 | 1 | 15 | 58 | 6 | 6 | 1 | 2 | 1 |  |
| White bread | 40 | 13 | 0 | 0 | 5 | 41 | 5 | 11 | 2 | 2 | 0 |  |
| Wholemeal bread | 20 | 11 | 2 | 2 | 28 | 38 | 4 | 11 | 2 | 2 | 0 |  |
| Brown, granary, wheatgerm bread | 17 | 13 | 3 | 2 | 36 | 29 | 4 | 12 | 2 | 2 | 0 |  |
| Broccoli | 22 | 4 | 2 | 27 | 20 | 26 | 6 | 4 | 5 | 4 | 6 |  |
| Cauliflower | 17 | 2 | 3 | 19 | 18 | 40 | 6 | 3 | 4 | 3 | 4 |  |
| Cucumber | 28 | 2 | 5 | 6 | 1 | 58 | 6 | 1 | 4 | 4 | 1 |  |
| Seeds (e.g. mixed seeds, sesame, sunflower, pumpkin, poppy, linseeds) | 15 | 13 | 1 | 10 | 28 | 33 | 6 | 3 | 2 | 3 | 2 |  |
| Unflavoured rice cakes, ice-cream wafer | 34 | 5 | 0 | 2 | 7 | 52 | 3 | 7 | 0 | 1 | 1 |  |

Table S5. Main foods contributing to each taste cluster

| Taste clusters | Products | Quantity and %Contribution |
| --- | --- | --- |
| Sweet-tasting foods | Snacks (biscuits, chocolates & candies) | 118 (17%) |
|  | Desserts (cakes, sweet pastries& pies) | 113 (16%) |
|  | Beverages (fizzy drinks, juices & alcoholic drinks) | 111 (16%) |
|  | Dairy products (fruit yogurt, ice cream & milk) | 100 (14%) |
|  | Fruits | 84 (12%) |
| Neutral-tasting foods | Potatoes | 60 (13%) |
|  | Bread | 58 (13%) |
|  | Unsalted butter & oils | 44 (10%) |
|  | Seafoods (white fish, shellfish & crab) | 37 (8%) |
|  | Vegetables | 31 (7%) |
| Savoury-tasting foods | Meats and poultry | 107 (31%) |
|  | Meat-based dishes & curries | 68 (20%) |
|  | Burgers & meat-based pastries | 64 (18%) |
|  | Vegetables and cheese-based foods | 45 (13%) |
|  | Seafoods (oily fish & sushi) | 16 (5%) |
| Salty-tasting foods | Snacks (crisps, biscuits & crackers) | 42 (37%) |
|  | Processed meat | 33 (29%) |
|  | Cheese | 22 (19%) |
|  | Canned/ready soups & pot noodle | 5 (4%) |
|  | Garlic bread | 3 (3%) |
| Bitter-tasting foods | Vegetables | 35 (45%) |
|  | Alcoholic drinks | 17 (22%) |
|  | Coffee & tea | 17 (22) |
|  | Dark chocolate & cocoa powder | 4 (5%) |
|  | Mustard & chilli papers | 4 (5%) |
| Sour-tasting foods | Fruits | 12 (31%) |
|  | Dipping and dressing | 12 (31%) |
|  | Plain yogurt, sour cream & buttermilk | 9 (23%) |
|  | Pickles + Viner | 5 (13%) |
|  | White wine | 1 (3%) |

Table S6. Characteristics of the adolescents’ dietary intakes as consumers and non-consumers of sour-tasting foods

|  | Weight of sour-tasting food consumed as percentage of the total food weight | | | | |
| --- | --- | --- | --- | --- | --- |
|  | **Non-consumers (n=214)** | **Consumers**  **(n=70)** | % Diff | Coeff. ^*^  (95%CI) | *P value* |
|  | Mean (95% CI) | |  |  |  |
| Energy (kcal/d) | 1592  (1527, 1657) | 1713  (1575, 1850) | 8% | 32 (-9, 72) | 0.12 |
| Carbohydrate (g/d) | 214 (204, 224) | 224 (206, 242) | 5% | 2 (-2, 7) | 0.37 |
| Protein (g/d) | 61 (58, 65) | 67 (61, 73) | 10% | 2 (-0.2, 3) | 0.08 |
| Fat (g/d) | 60 (57, 63) | 67 (60, 74) | 12% | 2 (-0.3, 4) | 0.09 |
| Total sugars (g/d) | 82 (76, 88) | 84 (75, 94) | 2% | 1 (-2, 3) | 0.59 |
| Free sugars (g/d) | 56 (50, 62) | 56 (8, 64) | 0% | 0.2 (-2, 3) | 0.84 |
| Fibre (g/d) | 15 (14, 15) | 16 (15, 18) | 7% | 0.2 (-0.2, 1) | 0.24 |
| Saturated fat (g/d) | 22 (21, 24) | 24 (21, 28) | 9% | 1 (-0.5, 2) | 0.27 |
| Sodium (mg/d) | 1795  (1690, 1900) | 1890  (1691, 2088) | 5% | 25 (-34, 83) | 0.40 |
| Fruit (g/d) | 61 (49, 73) | 76 (57, 95) | 25% | 5 (-0.3, 10) | 0.06 |
| Fruit Juice (g/d) | 83 (57,109) | 98 (63, 132) | 18% | 6 (-6, 18) | 0.29 |
| Brassica vegetables (g/d) | 10 (7, 13) | 16 (10, 23) | 60% | 2 (0.5, 4) | 0.01 |
| Other vegetables (g/d) | 82 (72, 92) | 101 (83, 120) | 23% | 1 (-5, 6) | 0.79 |
| Meat & poultry (g/d) | 59 (51, 66) | 67 (5, 83) | 14% | 5 (1, 9) | 0.02 |
| Processed meats (g/d) | 25 (21, 30) | 25 (18, 31) | 0% | -1 (-3, 1) | 0.41 |
| Cheese (g/d) | 18 (15, 21) | 20 (15, 24) | 11% | -0.2 (-2, 1) | 0.67 |

^*^ Change in nutrient/food per % increase in taste
